# Interventions in sports settings to reduce risky alcohol consumption and alcohol-related harm: a systematic review

**DOI:** 10.1186/s13643-016-0183-y

**Published:** 2016-01-21

**Authors:** Melanie Kingsland, John H. Wiggers, Khanrin P. Vashum, Rebecca K. Hodder, Luke Wolfenden

**Affiliations:** School of Medicine and Public Health, The University of Newcastle, Callaghan, New South Wales 2308 Australia; Hunter New England Population Health, Locked Bag 10, Wallsend, New South Wales 2287 Australia

## Abstract

**Background:**

Elevated levels of risky alcohol consumption and alcohol-related harm have been reported for sportspeople and supporters compared to non-sporting populations. Limited systematic reviews have been conducted to assess the effect of interventions targeting such behaviours.

**Methods:**

A review was undertaken to determine if interventions implemented in sports settings decreased alcohol consumption and related harms. Studies were included that implemented interventions within sports settings; measured alcohol consumption or alcohol-related injury or violence and were either randomised controlled trials, staggered enrolment trials, stepped-wedged trials, quasi-randomised trials, quasi-experimental trials or natural experiments. Studies without a parallel comparison group were excluded. Studies from both published and grey literature were included. Two authors independently screened potential studies against the eligibility criteria, and two authors independently extracted data from included studies and assessed risk of bias. The results of included studies were synthesised narratively.

**Results:**

The title and abstract of 6382 papers and the full text of 45 of these papers were screened for eligibility. Three studies met the inclusion criteria for the review. One of the included studies was a randomised controlled trial (RCT) of a cognitive-behavioural intervention with athletes within an Olympic training facility in the USA. The study reported a significant change in alcohol use between pre-test and follow-up between intervention and control groups. The other two studies were RCTs in community sports clubs in Ireland and Australia. The Australian study found a significant intervention effect for both risky alcohol consumption at sports clubs and overall risk of alcohol-related harm. The Irish study found no significant intervention effect.

**Conclusions:**

A limited number of studies have been conducted to assess the effect of interventions implemented in sports settings on alcohol consumption and related harms. While two of the three studies found significant intervention effects, it is difficult to determine the extent to which such effects are generalisable. Further controlled trials are required in this setting.

**Systematic review registration:**

PROSPERO CRD42014001739

**Electronic supplementary material:**

The online version of this article (doi:10.1186/s13643-016-0183-y) contains supplementary material, which is available to authorized users.

## Background

Alcohol consumption is a causal component of more than 200 diseases, injuries and other health conditions [1]. The diseases and conditions to which alcohol contributes are diverse due to multiple mechanisms of effect including the toxic effects on tissues and organs; dependence and subsequent lack of self-control over drinking behaviour and intoxication, whereby physical coordination, perception, cognition, consciousness and behaviour are impaired [2–4]. Worldwide, excessive alcohol consumption is responsible for 5.9 % of deaths and 5.1 % of the global burden of disease (as measured in disability adjusted life years (DALYs)) [4]. In 2012, this equated to approximately 3.3 million deaths and 139 million DALYs [4]. Such alcohol-related harm not only affects the user [4] but other people who have contact with the user such as family, friends and co-workers [5, 6] and society at large [3, 7, 8].

For most alcohol-related harms, there is a dose-response relationship between the amount (volume) of alcohol consumed and the risk of harm [9]. Consistent with such evidence, governments around the world have issued guidelines on the maximum amount of alcohol that is recommended to be consumed in order to reduce the harmful effects of alcohol in both the short (e.g. injury when intoxicated) and longer term (e.g. liver cirrhosis due to alcohol toxicity) [10].

People who engage in sports, either as players or fans, are more likely to consume alcohol at levels that put them at risk of short- and long-term harm compared to people not involved in sports. For instance, Weschler et al. 1997 found that in the USA, frequent heavy episodic drinking (≥3 heavy drinking episodes in the past 2 weeks) was more common amongst college student athletes (males 29 %; females 24 %) than students who were not athletes (males 18 %; females 15 %) [11]. Nelson et al. (2001) also found that a significantly higher proportion of US college athletes (57 %) reported binge drinking (≥5drinks) compared to students who were not athletes (49 %) [12]. Similar high levels of risky consumption have been found more recently amongst amateur and professional sportspeople in countries including Australia [13], Ireland [14], New Zealand [15, 16] and Brazil [17]. For instance, in New Zealand, O’Brien et al. 2005 found greater rates of binge drinking (≥6 drinks at least weekly) amongst sportspeople at the international/country elite level (59 %), provincial elite level (56 %) and non-elite level (51 %) compared to non-sportspeople (31 %) [16]. Similarly, a survey of Australian Football League (AFL) players by Dietze et al. (2008) found that the proportion of players that reported risky/high risk drinking for short-term harm (≥7 drinks on any 1 day on a monthly basis) ranged from 51 % during the playing season to 88 % at the end of the playing season, a proportion significantly greater than that for males in the general population (44 %) [18]. Higher levels of alcohol consumption have also been reported amongst spectators/fans when they are at games compared to when not at games and compared to non-spectator populations [19, 20].

Such heavy episodic drinking by sportspeople and fans has resulted in increased levels of alcohol-related harm amongst these population groups. In a recent systematic review, 10 of 11 included studies found higher rates of alcohol-related aggression and violence in sporting populations compared to non-sporting populations. Increased levels of aggression or violence amongst sports players and spectators were reported among middle/high school students, college/university students, current/former athletes and general adult populations at both elite and non-elite levels [21]. For instance, in a study by O’Brien et al. (2012), university sportspeople in Australia were over one and half times more likely than non-sportspeople to display aggressive behaviours such as verbal insults or assaults when intoxicated (*OR* 1.65; 95 % CI 1.19, 2.28) and almost twice as likely to damage property (*OR* 1.98; 95 % CI 1.38, 2.84) [22]. Similarly, in a study of amateur players of Gaelic football and hurling in Ireland, a significantly greater proportion of players reported getting in a fight due to their drinking (32 %) compared with a national sample of men of similar age (15 %) [14].

Given the disproportionate amount of alcohol-related harm experienced by people involved with sports, interventions to address excessive alcohol use within the sports setting have been recommended to reduce alcohol-related harm internationally by the World Health Organisation [23], governments [24] and experts [25].

To our knowledge, only two systematic reviews have been conducted assessing interventions targeting alcohol consumption and related harms in the sports setting [26, 27]. The first of these sought to review controlled trials of policy-based interventions in sporting organisations [26] but did not find any studies that met the inclusion criteria. The review included studies up until May 2007, those with a policy-focused intervention and an alcohol consumption outcome [26]. The second review [27] of alcohol harm reduction interventions in sports settings included five studies, all which were cross-sectional studies of the same multiple-component alcohol management intervention in community sporting clubs within Australia. The included studies reported significant improvements in alcohol consumption [28, 29], drink-driving [30, 31] and club revenue [32]. The review was limited to papers that were published in English and published in peer-reviewed journals, potentially excluding studies included in theses and dissertations, and other grey literature, such as government reports and unreported findings from studies reported in trial registries. The review was also limited to interventions that targeted adult populations of workplace employees or athletes, potentially excluding studies with adolescent populations and other people attending sporting venues, such as fans. Finally, the review did not conduct any assessment of the methodological quality of the included studies [33].

### Objectives

This review sought to determine if interventions implemented in the sports setting are effective relative to a comparison group in:

1. Reducing alcohol consumption at the sporting venue and/or overall alcohol consumption,
2. Reducing excessive alcohol consumption or intoxication at the sporting venue and/or overall excessive alcohol consumption or intoxication, and
3. Reducing alcohol-related violence or injury at the sporting venue and/or overall alcohol-related violence or injury.

## Methods

The review was undertaken according to the methods prescribed in the Cochrane Handbook for Systematic Reviews of Interventions [33] and is reported according to the Preferred Reporting Items for Systematic Reviews and Meta-analyses (PRISMA) [34]. A full report of the methods is available in the published review protocol [35].

### Eligibility criteria for included studies

#### Interventions

Studies were eligible to be included in the review if the intervention was implemented in a sporting setting and aimed to modify alcohol consumption behaviour and/or alcohol-related intoxication and/or or alcohol-related violence or injury. Interventions that aimed to address such outcomes, but also aimed to modify one or more additional health risk behaviour, were also eligible. Interventions could include, but were not limited to, health promotion, health education, regulatory or environmental initiatives. Interventions with a treatment focus were excluded. Sports settings were defined as settings (e.g. arenas, stadiums, grounds, complexes or ovals) where an organised sporting event or activity occurred either at a professional (elite) or non-professional (amateur/community) level, including competition games, training sessions or other club or team events.

#### Comparisons

Studies were eligible for inclusion in the review if they included a no intervention, attention or waitlist control group or an alternative intervention.

#### Primary outcomes

Studies with the following primary outcome measures were eligible for inclusion in the review: (1) alcohol consumption, such as number of drinks consumed or alcohol consumed at excessive/risky levels; (2) alcohol-related intoxication or (3) alcohol-related violence or injury. These measures could be assessed by any method, including surveys, observations, biochemical measures or police or medical records.

#### Study design

Studies with the following study designs were eligible to be included in the review: randomised controlled trials, including cluster randomised controlled trials; staggered enrolment trials [36] or stepped-wedged trials [37]; quasi-randomised trials, where group allocation is not purely random [38, 39]; quasi-experimental trials with comparison/control groups, including non-randomised pre-post (before-after) trials with one or more intervention and control groups [40], time-series/interrupted time-series trials (including multiple baseline trials) with independent control groups [36, 40], preference trials [37] and regression discontinuity trials [36] and natural experiment studies that have a comparison group [41]. Any trials without parallel comparison or control groups were excluded.

There was no eligibility criteria based on the length of follow-up, year of study publication, language, study publication status or study source (e.g. grey literature).

### Information sources and search strategy

Based on the abovementioned study eligibility criteria, a search strategy was developed and executed across the following electronic databases on the 20th August 2015: the Cochrane Central Registry of Controlled Trials (CENTRAL) (1974-); The Cochrane Library (1992-); MEDLINE (1946-); EMBASE (1947-); PsychINFO (1806-); SPORTDiscus (1985-); Dissertations and Theses (1743-); ERIC (1966-); PsycEXTRA (1908-) and CINAHL (1937-) (See Additional file 1).

The following sources were also searched (completed on 27th August 2015):

1. The contents of the peer-reviewed journals ‘Addiction’, ‘Journal of Studies on Alcohol and Drugs’ and ‘Medicine and Science in Sports and Exercise’ for the period May 2009–August 2015,
2. The first 200 citations from a Google Scholar search using search terms ‘alcohol’ AND ‘sport’ AND ‘program/programme OR intervention OR strategy’,
3. The results of searches of trial registries and the following topic relevant internet databases using the search terms ‘alcohol’ AND ‘sport’ AND ‘program/programme OR intervention OR strategy’—Alcohol and Alcohol Problems Science Database [http://etoh.niaaa.nih.gov/]; BiblioMap [http://eppi.ioe.ac.uk/webdatabases/Intro.aspx?ID=7]; Lifestyle Information Network [http://lin.ca/recreation-database]; SportScan Article Database [http://www.ausport.gov.au/information/archived/catalogue/nsic_catalogue]), and
4. The reference lists of included studies.

The authors of included trials were also contacted via email and asked to nominate any relevant trials.

### Study selection/screening

Following the removal of duplicate papers, two review authors independently screened the titles and abstracts of all papers identified through the search described above, assessing study eligibility using a standardised, pre-piloted screening tool and Endnote (version X7.0). Papers that did not meet the eligibility criteria were excluded. The same two reviewers independently examined the full text of all papers that were either deemed eligible or for which eligibility was uncertain. Any differences between reviewers in determining study eligibility were resolved by consensus and consultation with a third reviewer. Reasons for study ineligibility were recorded for all full-text papers and are described in Table 1. For papers where there was insufficient information to determine eligibility, the study authors were contacted for clarification. Review authors were not blinded to the name or institution of study authors or to journal titles.

**Table 1:** Reasons for exclusion of studies after full-text review

Study (author/s, year)	Reason for exclusion
Bagnardi et al. 2011 [52]	Intervention is not in a sports setting
Blaszczynski, 2011 [53]	No participants (commentary)
Bormann and Stone, 2001 [54]	No comparison group
Caetano et al. 2012 [55]	No participants (editorial)
Casswell and Gilmore, 1989 [56]	Intervention is not in a sports setting
Clarkson et al. 2002 [57]	No comparison group
Donohue et al. 2013 [58]	No participants (review paper)
Duff and Munroe, 2007 [59]	Does not report an outcome of interest
Finch and Donaldson, 2010 [60]	No participants (framework development)
Fromme et al. 1994 [61]	Intervention is not in a sports setting
Gregory, 2001 [62]	Intervention is not in a sports setting
Holder and Wagenaar, 1994 [63]	Intervention is not in a sports setting
Kelly, 2011 [64]	Does not report an outcome of interest
Kingsland et al. 2015 [65]	Does not report an outcome of interest
Maclean and Bonington, 2008 [66]	No participants (commentary)
Mann and Wickens, 2012 [67]	No participants (commentary)
Marcello et al. 1989 [68]	Intervention is not in a sports setting
Mentha and Waterman, 2009 [69]	No comparison group
O’Farrell et al. 2010 [14]	No comparison group
Pridemore et al. 2013 [70]	No comparison group
Reboussin et al. 2012 [71]	No comparison group
Rooney, 1984 [72]	No comparison group
Rossow and Norstrom, 2012 [73]	Intervention is not in a sports setting
Rowland et al. 2012a [29]	No comparison group
Rowland et al. 2012b [31]	No comparison group
Rowland et al. 2012c [74]	Cross-sectional study design
Rowland et al. 2012d [30]	Cross-sectional study design
Schewe et al. 1984 [75]	Does not report an outcome of interest
Shakeshaft et al. 2014 [76]	Intervention is not solely in a sports setting
Stuart et al. 2013 [77]	Intervention is not in a sports setting
Thombs, 2002 [78]	Intervention is not in a sports setting
Tricker, 1996 [79]	Intervention is not in a sports setting
Trolldal et al. 2013 [80]	No comparison group
Wagenaar et al. 2000 [81]	Community-wide intervention not specifically in a sports setting
Warpenius et al. 2010 [82]	Community-wide intervention not specifically in a sports setting
Watten, 1995 [83]	Intervention is not in a sports setting
Yoruk, 2014 [84]	Intervention is not in a sports setting

### Data extraction

Two review authors independently extracted data from eligible studies. A pre-piloted form based on the data extraction form in the Cochrane Handbook for Systematic Reviews of Interventions [33] was used to extract data for both evidence synthesis and assessment of study quality. Extracted information included authors; study setting (including country, type of sport and level of professionalism); study population and participants demographics (including age, gender and role, such as player or spectator/fan); study design; intervention and control conditions; trial outcomes and results and information for the assessment of study bias. As per the review protocol [35], it was anticipated that there may be a range of outcome measures across studies due to wide variation in measures of alcohol consumption, intoxication and harm. As such, no specific outcome measures were pre-specified. Where there were multiple papers reporting on the one study, the papers were grouped together and relevant information from across the papers used to complete the one data extraction form. Disagreement regarding data extraction was resolved through discussion between the two reviewers and, if required, consultation with a third reviewer. Study authors were contacted for data that were not available from the paper. Review authors that were extracting data were not blinded to the name or institution of study authors or to journal titles.

Upon finalisation of the data extraction process, one review author transferred study information into the included studies (Table 2) and a second review author checked the data.

**Table 2:** Characteristics of included studies

Study	Study design/setting	Participants	Intervention and control conditions	Outcomes of interest to the review
Carr 1992 [42]	Individually randomised trial Olympic Training Centre (OTC) in Colorado Springs, Colorado, USA.	Eight resident teams of elite athletes representing archery, gymnastics, shooting, table tennis and handball. Intervention group: 30 athletes; control group: 23 athletes Age: mean 21.3 (SD 4.1) Gender: males 59 %, females 41 % Inclusion criteria: athletes in resident training programmes at the OTC.	Intervention condition: multimodal substance abuse programme based on a cognitive-behavioural model, which included: • education component (2.5 h) • decision-making/coping skills component (3 h) • social skills/self-esteem component (2.5 h). Each component included lecture presentations, group discussions, role-play exercises, and written materials. Control condition: no intervention. Intervention offered after trial period.	All outcomes were assessed at pre-test, post-test and at the end of a 7-week follow-up period via self-completed questionnaires. • Frequency of use of alcohol in the last month • Change score for frequency of use in alcohol in the last month from pre-test to follow-up, coded as: 1 = decrease in use 2 = no change 3 = increase
Kingsland et al. 2015 [44]	Cluster randomised controlled trial Non-elite, community football clubs within the Hunter, New England and Sydney regions of New South Wales, Australia	Eighty-eight football clubs (rugby league, rugby union, soccer/association football and Australian rules football) and club members. Intervention group: 43 clubs; 700 members at pre-intervention cross section; 567 members post-intervention cross section. Control group: 45 clubs; 711 members pre-intervention cross section; 577 members post-intervention cross section. Members pre-intervention: Age: average 30 years+ Gender: intervention group 77.4 % male; control group 87 % male) Role: intervention group 60 % players, 26 % members/supporters, 14 % officials; control group 47 % players, 36 % members/supporters, 17 % officials. Inclusion criteria: Clubs: community level, non-elite football clubs who had over 40 members, sold alcohol and were not participating in an alcohol management improvement program. Members: club member who were 18+ years and spoke English	Intervention condition: 2.5 year accreditation programme which included: • Adherence to liquor licence requirement in terms of signage and alcohol service hours and areas • Staff trained in responsible service of alcohol • Water and substantial food is provided • Intoxicated people not permitted to enter, not served alcohol and not permitted to remain at the club • Alcoholic drinks are only served in standard drink measures • Club maintains a register of alcohol-related incidents • Bar servers do not consume alcohol • Non-alcoholic drinks and low-alcoholic drinks are available and are cheaper than full-strength alcoholic drinks • Club does not serve ‘shots’ or double-nips of alcohol or ready-to-drink products over 5 % alcohol/volume • Club does not conduct drinking games/promotions that encourage risky alcohol consumption • Club has some sponsorship that is not from the alcohol industry • Club has developed a written alcohol management policy and distributed it to members. *Implementation supports:* based on theoretical frameworks for organisational change and consisted of: project officer support, implementation cost recovery, accreditation and associated merchandise, printed resources and newsletters, observational audits and feedback online training and letters of support from state sporting organisation. Control condition: control (and intervention) clubs were given printed resources on topics unrelated to the trial outcomes.	All outcomes were assessed at pre- and post-intervention using self-reported measures collected via telephone survey. • Risky alcohol consumption defined as ≥5 standard drinks on the one occasion • Alcohol Use Disorders Identification Test (AUDIT): ▪ Median total AUDIT score ▪ Total AUDIT score of ≥8 (indicative of hazardous consumption) ▪ Alcohol consumption subscale (score ≥6 for items 1–3) ▪ Dependency subscale (score of ≥4 for items 4–6) ▪ Alcohol-related problems subscale (score ≥1 for items 7–10). All outcome analyses adjusted for clustering and pre-intervention values.
O’ Farrell 2010 [43]	Cluster randomised controlled trial Gaelic Athletic Association (GAA) amateur sporting clubs in the Republic of Ireland.	Forty-one hurling, Gaelic football and handball clubs within two counties in Ireland and club players. Intervention group: 12 clubs; 332 members at pre-intervention; 218 members post-intervention. Control group: 29 clubs; 628 members pre-intervention; 441 members post-intervention. Players pre-intervention: Mean age: 24 years Gender: All male Inclusion criteria: clubs: within two study counties in Ireland Players: uninjured GAA male club players aged 16 years and above.	Intervention condition: Community mobilisation approach targeting the club environment and individual player behaviour implemented over four months. Intervention included: • Alcohol education for the players (1x50mins) • Alcohol education for coaches (1x40mins) • Alcohol policy training for club managers and coaches (1 × 40 min) • Alcohol information media campaign *Implementation supports*: health promotion staff, presentation materials, handouts and advertising materials. Control condition: control (and intervention clubs) received an education session on sports nutrition.	All outcomes were assessed at pre- and post-intervention using self-reported measures via paper questionnaires: • Alcohol use disorder identification test (AUDIT): ▪ Mean total AUDIT score ▪ Total AUDIT score of ≥ 8 ▪ AUDIT hazardous alcohol use subscale (score ≥6 for items 1–3) ▪ AUDIT dependency subscale (score ≥4 for items 4–6) ▪ AUDIT harmful alcohol use subscale (score ≥1 for items 7–10). • Yearly alcohol consumption (litres of pure alcohol) • ≥21 standard drinks per week • Binge drinking (≥6 drinks one sitting) • Mean alcohol-related harms score (of total of 13)

### Assessment of risk of bias

Two review authors independently assessed risk of bias in eligible studies by assessing the adequacy of study characteristics as outlined in the Cochrane Handbook for Systematic Reviews of Interventions [33]. Disagreement regarding assessment of risk of bias was resolved through discussion between the two reviewers and, if required, consultation with a third reviewer.

For any non-randomised trials, the authors planned to assess selection bias that may have led to confounding of the outcome of interest and the appropriateness of any statistical methods used to adjust for such confounding. Additional biases specific to individual study designs were planned to be assessed on a case-by-case basis and in consultation with relevant methodological experts and noted in a supplementary risk of bias table.

### Data synthesis and analysis

Intervention effects for the relevant outcomes of all included studies were described narratively. It was planned that Review Manager (RevMan Review Manager (RevMan) [Computer program] Version 5.2. Copenhagen: The Nordic Cochrane Centre, The Cochrane Collaboration, 2012) be used to undertake meta-analyses using a random effects model where outcome data reported by included trials enabled pooling and where trials were sufficiently homogeneous in terms of participants, interventions and outcome characteristics. For cluster randomised controlled trials (RCTs), meta-analyses were planned to be performed using adjusted effect size estimates and standard errors using the generic inverse variance method in Revman. For cluster trials where suitable adjusted effects were not reported, an effective sample size was planned to be calculated using the intra-class correlations provided in study reports.

#### Assessment of study heterogeneity

It was planned that heterogeneity between studies be assessed using both visual inspection of forest plots and the *I*^2^ statistic. An *I*^2^ value greater than 50 % was planned to be considered indicative of substantial heterogeneity.

## Results

### Results of the search

The searches generated 6382 papers (following duplicate removal). Screening of titles and abstracts identified 45 papers for full-text review (see Additional file 2: PRISMA flowchart). Of these, three trials (Carr 1992 [42]; O’Farrell 2010 [43]; Kingsland et al. 2015 [44]) met the inclusion criteria.

### Excluded studies

Of the 45 papers for which the full text was examined, 37 were deemed ineligible (Additional file 2: PRISMA flowchart). Six were deemed ineligible based on participants, four based on outcome, 10 based on having no parallel comparator, 15 based on intervention and two based on study design (Table 1).

### Characteristics of included studies

A description of the included trials is presented in Table 2. In the Carr 1992 trial, individual athletes residing at a training facility in the USA were randomised to control (*n* = 23) and intervention (*n* = 30) groups [42]. The trial intervention was based on a cognitive-behavioural model and was delivered in three separate sections: (1) an education component on the effects of substance misuse (2.5 h); (2) a decision-making/coping skills component (3 h) and (3) a social skill/self-esteem component (2.5 h). Each component included lecture presentations, group discussion, role-play exercises and written materials. The control group received no intervention during the trial period. Trial outcomes were assessed pre-intervention, immediately post-intervention and at a 7-week post-intervention follow-up via self-administered questionnaires and included a measure of how often alcohol was used in the last month [42].

The study by Kingsland et al. (2015) was a cluster randomised controlled trial, with 42 community-level, non-elite football (Australian Rules, Soccer/Association Football, Rugby League and Rugby Union) clubs randomly allocated to the intervention group and 45 such clubs allocated to the control group [44]. The 2.5-year intervention involved the participating clubs implementing responsible alcohol management strategies, including reduced pricing of low and non-alcoholic drinks, responsible service of alcohol training of staff and restrictions on drinking games and promotion that encourage rapid intoxication. The control clubs received written materials unrelated to the outcome measures. Repeat cross-sectional surveys of players, supporters and officials were employed at pre- and post-intervention to measure trial outcomes. The trial outcomes included risky alcohol consumption (≥5 drinks on the one occasion) at least once a month at the sports club and the following measures of overall alcohol consumption risk: median total Alcohol Use Disorders Identification Test (AUDIT) score; total AUDIT score of ≥8; AUDIT alcohol consumption/hazardous use subscale score of ≥6; AUDIT dependence subscale score of ≥4 and AUDIT alcohol-related problems/harmful use subscale score of ≥1. Data were collected from 1411 participants pre-intervention and 1144 participants post-intervention [44].

The study by O’Farrell (2010) involved a cluster-controlled trial in Gaelic football and hurling clubs. All sports clubs in one county within Ireland acted as control clubs (*n* = 29) and randomly selected clubs within another county participated in the trial as intervention clubs (*n* = 12) [43]. The two participating counties were selected on a convenience basis. Intervention strategies were implemented over a 4-month period and involved strategies at the community level (e.g. media campaign), club level (e.g. responsible alcohol service practices) and player level (e.g. education). Repeat cross-sectional surveys were used at pre- and immediately post-intervention to measure trial outcomes. Trial outcomes included yearly alcohol consumption (litres of pure alcohol); consumption of ≥21 standard alcoholic drinks per week; binge drinking (≥6 standard alcoholic drinks in one sitting); mean total AUDIT score; total AUDIT score of ≥8; AUDIT alcohol consumption/hazardous use subscale score of ≥6; AUDIT dependence subscale score of ≥4 and AUDIT alcohol-related problems/harmful use subscale score of ≥1. The study also included a mean alcohol-related harm score as a measure of overall alcohol-related violence or injury. Data were collected from 960 players pre-intervention and 659 post-intervention [43].

For all trials, alcohol harm reduction was the primary aim and focus of the intervention.

### Risk of bias in included studies

**Fig. 1:**
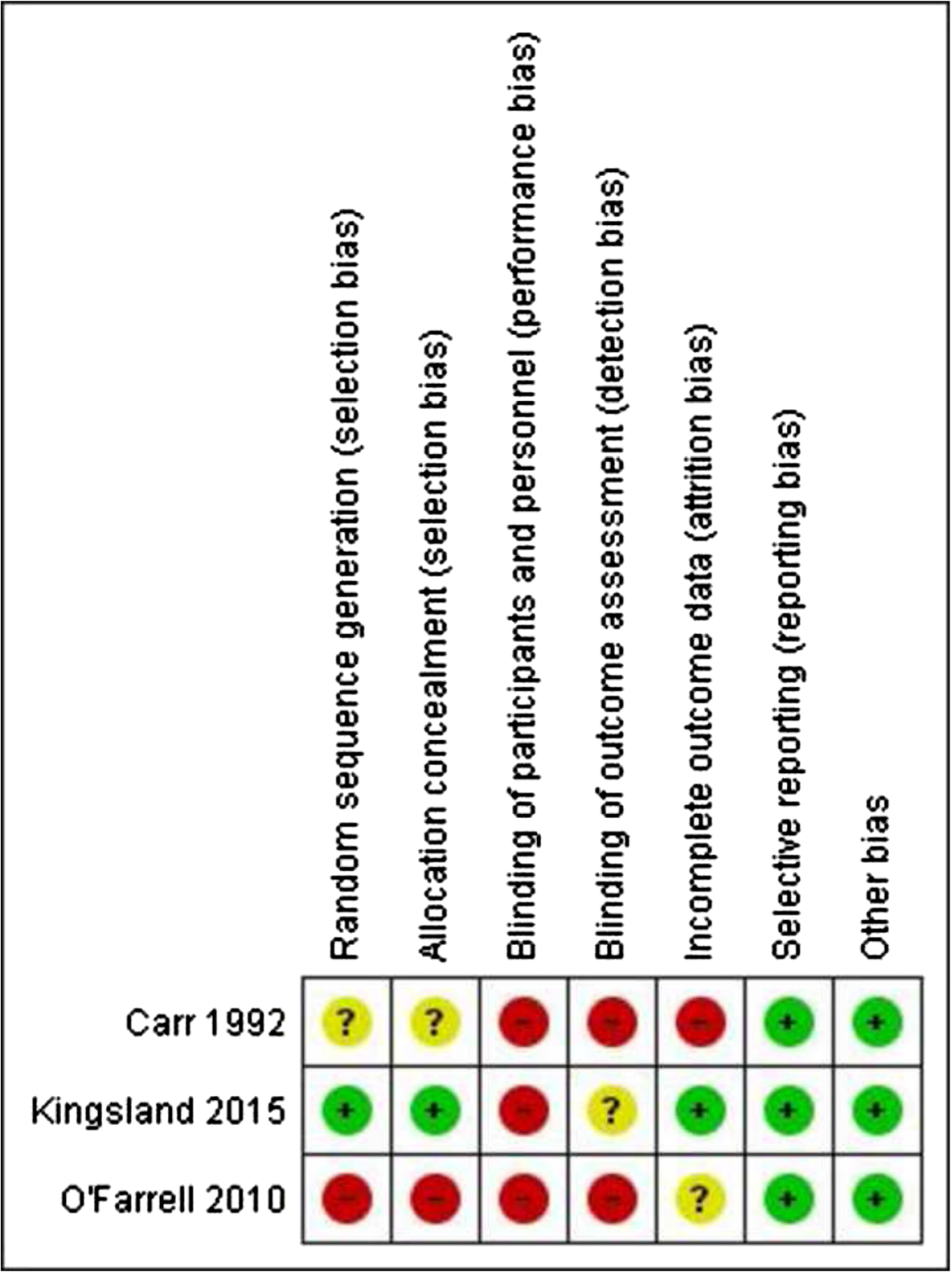
Risk of bias summary: review authors’ judgments about each risk of bias item for each included study

**Fig. 2:**
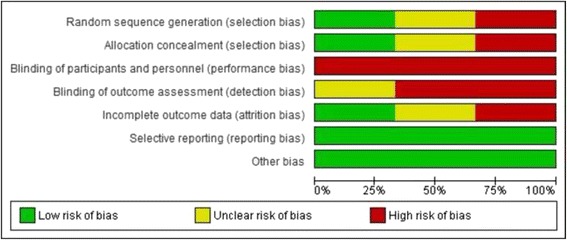
Risk of bias graph: review authors’ judgments about each risk of bias item presented as percentages across all included studies

The level of risk of bias is presented separately for each study in Fig. 1 and as a combined study assessment of risk of bias in Fig. 2. Additional file 3 contains justification for each risk assessment.

Risk of selection bias differed across studies. Only Kingsland et al. 2015 [44] reported appropriate random sequence generation and club allocation concealment methods and, therefore, was assessed as having a low risk of selection bias. All included studies were public health interventions in which it was not possible for participants or study personnel to be blind to group allocation, and therefore, risk of performance bias was deemed to be high for all studies. For all studies, all outcome measures were self-reported by participants and subsequently detection bias was deemed to be high for two out of three studies. For Kingsland et al. 2015 [44], such risk was deemed to be unknown because while club members who self-reported study outcomes were not intentionally blinded from knowing which group their club was allocated to, their knowledge of this is unknown and the research personnel collecting outcome data by telephone surveys were blind to group allocation. Risk of attrition bias differed across studies. Only Kingsland et al. 2015 [44] reported undertaking intention-to-treat analysis and therefore scored low in regard to risk of attrition bias. For all studies, risk of reporting bias was deemed to be low as either all planned outcomes were reported or explanation provided as to why this was not the case. For the two cluster trials [43, 44], the potential risk of bias due to sports club member/player selection was assessed. For both studies, risk of such selection bias was deemed to be low due to the use of either a quasi-random or census approach.

### Effect of intervention

The intervention effects of individual studies are summarised in Table 3.

**Table 3:** Intervention effects of included studies

Study	Intervention effects on outcomes of interest to the review
Carr 1992	Frequency of alcohol use No significant difference was reported between the groups at pre-test (*X* ^2^ = 5.94, *P* = 0.20), post-test (*X* ^2^ = 5.48, *P* = 0.24) or follow-up (*X* ^2^ = 5.96, *P* = 0.20). Change score Significant difference between the treatment and control group at follow-up (*X* ^2^ = 6.42, *P* < 0.05). In the treatment group, 3.6 % reported decreased use, 89.3 % no change and 7.1 % increased use. In the control group, 21.1 % reported decreased use, 57.9 % no change and 21.1 % increased use.
Kingsland et al. 2015	Risky alcohol consumption At baseline, 27 % of intervention club and 25 % of control club members reported risky alcohol consumption. Post-intervention, 19 % of intervention club members reported risky alcohol consumption compared to 24 % of control club members (OR = 0.63 95 % CI 0.40–1.00, *P* = 0.05). Median total AUDIT score (min, max) Pre-intervention: control 7 (0, 26), intervention 8 (0, 28); post-intervention: control 7 (0, 25), intervention 6 (0, 26) (*P* < 0.01). Total AUDIT score ≥8 Pre-intervention: control 46 %, intervention 54 %; post-intervention: control 45 %, intervention 38 % (OR = 0.58 (95 % CI 0.38–0.87, *P* < 0.01). AUDIT alcohol consumption subscale Pre-intervention: control 57 %, intervention 61 %; post-intervention: control 55 %, intervention 47 % (OR = 0.60 95 % CI 0.41–0.87 *P* value <0.01). AUDIT alcohol dependence subscale Pre-intervention: control 3 %, intervention 4 %; post-intervention: control 4 %, intervention 1 % (OR = 0.20 95 % CI 0.06–0.65 *P* value <0.01). Alcohol-related problems subscale Pre-intervention: control 48 %, intervention 56 %; post-intervention: control 45 %; intervention 41 % (OR = 0.67 95 % CI 0.43–1.03 *P* value 0.03).
O’ Farrell 2010	Mean total AUDIT score Post-intervention: control 11.0 (95 % CI 10.4–11.7); intervention 11.0 (95 % CI 10.0–11.4); *P* = 0.94. Total AUDIT score ≥8 Post-intervention: control 69.9 % (95 % CI 64.1–76.8), intervention: 72.2 (95 % CI 63.7–80.6); *P* = 0.66. AUDIT hazardous alcohol use subscale Post-intervention: control 95.1 % (95 % CI 92.6–97.6); intervention 95.0 % (95 % CI 91.5–98.6); *P* = 0.97. AUDIT dependency subscale Post-intervention: control 60.5 % (95 % CI 53.2–67.8), intervention 59.7 % (95 % CI 49.2–70.1); *P* = 0.90. Harmful alcohol use subscale Post-intervention: control 68.5 % (95 % CI 63.1–73.8), intervention 74.8 % (95 % CI 67.1–85.6); *P* = 0.17. Mean yearly consumption Post-intervention: control 11.6 L (95 % CI 9.2–14.2), intervention 8.8 L (95 % CI 5.6–12.1); *P* = 0.17. ≥21 standard drinks per week Post-intervention: control 28.5 % (95 % CI 21.4–35.7), intervention 20.1 % (95 % CI 10.6–29.5); *P* = 0.15. Binge drinking Post-intervention: control 43.5 % (95 % CI 35.2–51.8), intervention 49.1 % (95 % CI 37.8–60.3); *P* = 0.42. Mean alcohol harm score Post-intervention: control 3.0 (95 % CI 2.5–3.6), intervention 2.5 (95 % CI 1.7–3.3); *P* = 0.26.

#### Reducing alcohol consumption at the sporting venue and/or overall alcohol consumption

Carr 1992 reported a significant difference between the intervention and control groups in change in alcohol use frequency between pre-test and follow-up (*X*^2^ = 6.42, *P* < 0.05) [42]. In the intervention group, 3.6 % reported decreased use, 89.3 % no change and 7.1 % increased use and, in the control group, 21.1 % reported decreased use, 57.9 % no change and 21.1 % increased use. There was no significant difference in frequency of alcohol use in the last month between the groups at pre-test (*X*^2^ = 5.94, *P* = 0.20), post-test (*X*^2^ = 5.48, *P* =0.24) or follow-up (*X*^2^ = 5.96, *P* = 0.20) [42].

In the cluster RCT by O’Farrell (2010), there was no significant difference in mean yearly overall alcohol consumption (in any setting) at post-intervention between control (11.6 L; 95 % CI 9.2, 14.2) and intervention groups (8.8 L; 95 % CI 5.6, 12.1) (*P* = 0.17) [43]. Kingsland et al. (2015) did not report any such overall measures of alcohol consumption volume [44].

#### Reducing excessive alcohol consumption or intoxication at the sporting venue and/or overall excessive alcohol consumption or intoxication

Kingsland et al. 2015 was the only trial that reported an outcome related to excessive alcohol consumption at a sporting venue [44]. At baseline, 27 % of intervention club and 25 % of control club members reported consuming alcohol at risky levels (≥5 drinks) at their sports club. Post-intervention, a significantly smaller proportion of intervention club members (19 %) reported such a level of alcohol consumption at their sports club compared to control club members (24 %) (OR = 0.63 95 % CI 0.40–1.00, *P* = 0.05) [44].

Kingsland et al. 2015 [44] and O’Farrell 2010 [43] were the only studies that reported data measuring the impact of interventions on overall excessive alcohol consumption in any setting. Both studies used the same measures of alcohol-related harm (overall AUDIT score and AUDIT subscale scores), and these data were pooled. However, statistically heterogeneity was high (*I*^2^: 76–87 %).

Kingsland et al. 2015 found that a significantly lower proportion of intervention group club members (38 %) reported AUDIT scores above eight compared to control group club members (45 %), post-intervention (OR = 0.58 (95 % CI 0.38–0.87, *P* < 0.01) [44], whereas, O’Farrell did not find any significant difference between treatment groups for this measure (post-intervention: control 69.9 % (95 % CI 64.1–76.8, intervention: 72.2 % (95 % CI 63.7–80.6; *P* = 0.6)) [43].

Kingsland et al. (2015) reported significant intervention effect with respect to the AUDIT alcohol consumption subscale (post-intervention: control 55 %, intervention 47 % (OR = 0.60 95 % CI 0.41–0.87 *P* value <0.01)) and the AUDIT alcohol dependence subscale (post-intervention: control 4 %, intervention 1 % (OR = 0.20 95 % CI 0.06–0.65 *P* value <0.01)) [44]. In contrast, O’Farrell found no such effects (AUDIT alcohol consumption subscale, post-intervention: control 95.1 % (95 % CI 92.6–97.6); intervention 95.0 % (95 % CI 91.5–98.6) (*P* = 0.97); AUDIT dependency subscale: post-intervention: control 60.5 % (95 % CI 53.2–67.8); intervention 59.7 % (95 % CI 49.2–70.1) (*P* = 0.90)) [43].

Neither Kingsland et al. (2015) (post-intervention: control 45 %; intervention 41 % (OR = 0.67 95 % CI 0.43–1.03 *P* = 0.07) [44] nor O’Farrell (2010) found a significant intervention effect with respect to the AUDIT alcohol-related problems subscale (post-intervention: control 68.5 % (95 % CI 63.1–73.8), intervention 74.8 % (95 % CI 67.1–85.6) (*P* = 0.17)) [43].

Kingsland et al. 2015 found a significant difference in median AUDIT score between members of control and intervention group sports clubs post-intervention (control: 7 (range = 0, 25); intervention: 6 (range = 0, 26) (*P* < 0.01)) [44]. O’Farrell 2010 did not find any significant difference between intervention and control groups in the proportion of players reporting consumption of 21 or more standard drinks per week (post-intervention: control 28.5 % (95 % CI 21.4–35.7), intervention 20.1 % (95 % CI 10.6–29.5); *P* = 0.15), the proportion of players reporting consumption of six or more drinks in one setting (‘binge drinking’) (post-intervention: control 43.5 % (95 % CI 35.2–51.8), intervention 49.1 % (95 % CI 37.8–60.3); *P* = 0.42), mean total AUDIT score (post-intervention: control 11.0 (95 % CI 10.4–11.7); intervention 11.0 (95 % CI 10.0–11.4); *P* = 0.94) or mean alcohol harm score (post-intervention: control 3.0 (95 % CI 2.5–3.6), intervention 2.5 (95 % CI 1.7–3.3); *P* = 0.26) [43].

#### Reducing alcohol-related violence or injury at the sporting venue and/or overall alcohol-related violence or injury

No included studies reported separate, discrete measures of alcohol-related violence or injury.

Due to the heterogeneity of included studies, no quantitative data synthesis was undertaken.

## Discussion

Despite evidence demonstrating elevated levels of risky alcohol consumption and alcohol-related harm amongst people involved in sports and recommendations for the development of interventions to address this risk, the review identified only three controlled trials of relevant interventions within the sports setting. Two of the included studies reported a positive effect on one or more alcohol consumption or alcohol-related harm outcome either within the sports setting or overall [42, 44]. As none of the included trials reported discrete alcohol-related violence or injury outcomes, the impact of such interventions on injury or violence in sporting club contexts is unknown.

The findings of the study by Carr (1992) were equivocal as they indicate that while 7.1 % of the intervention group reported increased alcohol use compared with 21.1 % of the control group, 3.6 % of the intervention group reported decreased use of alcohol compared with 21.1 % of the control group [42]. As such, it is unclear whether the cognitive-behavioural intervention targeting substance use by athletes in training settings was effective in reducing alcohol misuse and related harms. The findings should also be considered in the context of a rating of high risk of performance bias, detection bias and attrition bias, which further supports the equivocal nature of the reported findings. Findings of reviews of similar interventions (social norm, motivational interviewing) in young adults in college/university and non-college settings have found no meaningful benefits associated with such interventions for the prevention of alcohol misuse [45, 46].

The two included randomised controlled trials that tested the impact of multicomponent alcohol harm reduction interventions in community sports clubs reported mixed results. The intervention reported by Kingsland et al. 2015 was effective in reducing risky alcohol consumption by club members within the community football club setting and the risk of alcohol-related harm to club members as measured by AUDIT [44], whereas the O’Farrell 2010 study found no intervention effect across all related outcomes [43]. These two studies differed in a number of ways. For example, the intervention in the Kingsland et al. (2015) trial [44] was implemented over 2.5 years whereas the O’Farrell (2010) trial [43] was implemented for 4 months. A longer implementation period potentially afforded the Kingsland et al. (2015) [44] trial both more time for the intervention to change alcohol-related harm outcomes, as well as a greater dose of intervention, both of which have been found to be associated with intervention effectiveness [47]. These findings should also be considered in the context of the risk of bias assessment. The O’Farrell (2010) [43] study was considered to have a potentially high risk of bias across four (selection bias x 2; performance bias; detection bias) of the seven items, whereas, the study by Kingsland et al. (2015) [44] was rated as high risk of bias for only performance bias. As such, the findings from the Kingsland et al. (2015) [44] study are potentially more reliable than those of O’Farrell (2010) [43].

No studies were found that examined the effectiveness of interventions in reducing alcohol consumption or alcohol-related harm by players or spectators in large sporting venues, such as arenas and stadia. This is despite evidence from Europe [48], the USA [49] and New Zealand [50] that suggests that sporting clubs and venues fail to implement alcohol management practices comprehensively and consistently. For instance, Drygas et al. reported that only 22 % of 88 sports stadiums across 10 European countries implemented initiatives to encourage responsible alcohol use [48], and Lenk et al. found that only 27 % of 66 professional sports stadiums in the USA implemented ‘11 or more’ of 12 alcohol control policies/practices [49]. The findings are also limited in their generalisability beyond the countries in which they were conducted, where the sporting venues, populations and cultures may differ. For instance, despite emerging data from countries including Brazil [17] and Japan [51] regarding elevated levels of alcohol-related harm amongst sporting populations, the potential to generalise the findings of studies from Ireland and Australia to such countries is unknown.

Compared to previous systematic reviews of interventions to reduce alcohol-related harm in the sports setting, this review included three controlled trials that had not been reported in previous reviews [26, 27], primarily due to the date range of the search and the inclusion of grey literature. The review conducted by Kolar et al. (2015) [27] included five cross-sectional studies on the same intervention, which was also the same intervention trialled by Kingsland et al. (2015) in the study included in this review. As reported by Kolar et al. (2015) [27], these five studies reported significant intervention effects in alcohol consumption [28, 29], drink-driving [30, 31] and club revenue [32] and, as such, further support the findings of Kingsland et al. (2015).

A number of potential methodological limitations of the review need to be noted. First, a design filter was used to manage the search, as is suggested for complex reviews of public health and health promotion interventions [33]. As such, the review needs to be considered in this context as, while it is considered unlikely, the inclusion of a design filter may have resulted in potentially eligible studies being missed. Second, some studies may have been missed through limitations of the databases searched and non-publication of studies with negative results.

Given the paucity of controlled trials of interventions in the sports setting that aim to reduce risky alcohol consumption and alcohol-related harm and the variable quality and findings of those that have been conducted, further high quality trials are required in order to determine if such interventions are broadly effective and should be further adopted by policy makers and sports administrators.

## Conclusions

A limited number of studies have been conducted to assess the effect of interventions implemented in sports settings on alcohol consumption and related harms. While two of the three studies found significant intervention effects, it is difficult to determine the extent to which such effects are generalisable. Further controlled trials are required in this setting that adhere to high standards of trial methodology, particularly in professional sports settings where there is currently an absence of such research trials.

## Additional files

Additional file 1:
**Results of database search.** This file contains the search strategy and results of the search executed across the databases described in the methods section. The search was undertaken at two time points to ensure all relevant studies were included. (DOCX 200 kb) Additional file 2:
**PRISMA flowchart.** This file contains the PRISMA flowchart for the review. The flowchart includes the number of studies screened, number of studies excluded and number of studies included in the review.(PDF 190 kb) Additional file 3:
**Risk of bias assessment: detailed results.** This file contains the detailed results of the risk of bias assessment of included studies. The reasons for each assessment rating are provided.(DOCX 21 kb)

## Abbreviations

AFL: Australian Football League
AUDIT: Alcohol Use Disorders Identification Test
DALYs: disability adjusted life years
PRISMA: Preferred Reporting Items for Systematic Reviews and Meta-analyses
RCT: randomised controlled trial

## References

[1] World Health Organisation (1992). WHO Statistical Classification of Diseases and Related Health Problems (ICD) 10th revision.

[2] World Health Organisation (2004). Global status report on alcohol 2004.

[3] World Health Organisation (2007). WHO expert committee on problems related to alcohol consumption. WHO technical report series.

[4] World Health Organisation (2014). Global status report on alcohol and health 2014.

[5] Laslett A, Room R, Ferris J, Wilkinson C, Livingston M, Mugavin J (2011). Surveying the range and magnitude of alcohol’s harm to others in Australia. Addiction.

[6] Connor J, Casswell S (2012). Alcohol-related harm to others in New Zealand: evidence of the burden and gaps in knowledge. N Z Med J.

[7] Nutt DJ, King LA, Phillips LD (2010). Drug harms in the UK: a multicriteria decision analysis. Lancet.

[8] Thavorncharoensap M, Teerawattananon Y, Jomkwan Y, Lertpitakpong C, Chaikledkaew U. The economic impact of alcohol consumption: a systematic review. Subst Abuse Treat Prev Policy. 2009;4(20):doi:10.1186/747-597X-4-20. 10.1186/1747-597X-4-20 PMC2791094 19939238

[9] Rehm J, Room R, Graham K, Monteiro M, Gmel G, Sempos C (2003). The relationship of average volume of alcohol consumption and patterns of drinking to burden of disease—an overview. Addiction.

[10] Furtwængler NAFF, de Visser RO (2013). Lack of international consensus in low-risk drinking guidelines. Drug Alcohol Rev.

[11] Wechsler H, Davenport AE (1997). Binge drinking, tobacco, and illicit drug use and. J Am Coll Heal.

[12] Nelson T, Wechsler H (2001). Alcohol and college athletes. Med Sci Sport Exerc.

[13] Black D, Lawson J, Fleishman S (1999). Excessive alcohol use by non-elite sportsmen. Drug Alcohol Rev.

[14] O'Farrell A, Allwright S, Kenny S, Roddy G, Eldin N (2010). Alcohol use among amateur sportsmen in Ireland. BMC Res Notes.

[15] O'Brien KS, Ali A, Cotter JD, O'Shea RP, Stannard S, O'Brien KS (2007). Hazardous drinking in New Zealand sportspeople: level of sporting participation and drinking motives. Alcohol Alcohol.

[16] O'brien KS, Blackie JM, Hunter JA (2005). Hazardous drinking in elite New Zealand sportspeople. Alcohol Alcohol.

[17] Bedendo A, Opaleye ES, Andrade ALM, Noto AR (2013). Heavy episodic drinking and soccer practice among high school students in Brazil: the contextual aspects of this relationship. BMC Public Health.

[18] Dietze PM, Fitzgerland JL, Jenkinson RA (2008). Drinking by professional Australian Football League (AFL) players: prevalence and correlates of risk. Med J Australia.

[19] Glassman T, Werch CE, Jobli E, Bian H (2007). Alcohol-related fan behavior on college football game day. J Am Coll Health.

[20] Nelson TF, Wechsler H (2003). School spirits: alcohol and collegiate sports fans. Addict Behav.

[21] Sønderlund AL, O’Brien K, Kremer P, Rowland B, De Groot F, Staiger P (2014). The association between sports participation, alcohol use and aggression and violence: a systematic review. J Sci Med Sport.

[22] O’Brien KS, Kolt GS, Martens MP, Ruffman T, Miller PG, Lynott D (2012). Alcohol-related aggression and antisocial behaviour in sportspeople/athletes. J Sci Med Sport.

[23] World Health Organisation (2011). Global status report on alcohol and health.

[24] National Preventative Health Taskforce. Australia the Healthiest Country by 2020. Technical Report No. 3. Preventing alcohol-related harm in Australia: a window of opportunity. Barton: ACT: Commonwealth of Australia; 2009.

[25] Kelly B, King L, Bauman AE, Baur LA, Macniven R, Chapman K (2014). Identifying important and feasible policies and actions for health at community sports clubs: a consensus-generating approach. J Sci Med Sport.

[26] Priest N, Armstrong R, Doyle J, Waters E. Policy interventions implemented through sporting organisations for promoting healthy behaviour change. Cochrane Database Syst Rev. 2008;3(Art. No.: CD004809. DOI: 10.1002/14651858.CD004809.pub3.). 10.1002/14651858.CD004809.pub3 PMC6464902 18646111

[27] Kolar C, von Treuer K (2015). Alcohol misuse interventions in the workplace: a systematic review of workplace and sports management alcohol interventions. Int J Mental Health Addict.

[28] Rowland B, Allen F, Toumbourou JW (2012). Association of risky alcohol consumption and accreditation in the ‘Good Sports’ alcohol management programme. Epidemiol Community Health.

[29] Rowland B, Allen F, Toumbourou J (2012). Impact of alcohol harm reduction strategies in community sports clubs: pilot evaluation of the good sports program. Health Psychol.

[30] Rowland B, Toumbourou J, Allen F (2012). Drink-driving in community sports clubs: adopting the good sports alcohol management program. Accident Anal Prev.

[31] Rowland B, Toumbourou J, Allen F (2012). Reducing alcohol-impaired driving in community sports clubs: evaluating the good sports program. J Stud Alcohol Drug.

[32] Crundall I (2012). Alcohol management in community sports clubs: impact on viability and participation. Health Promot J Austr.

[33] Higgins J, Green S. Cochrane Handbook for Systematic Reviews of Interventions Version 5.1.0 [updated March 2011]. 2011. www.cochrane-handbook.org. Accessed 9/8/2013.

[34] Moher D, Liberati A, Tetzlaff J, Altman DG. Preferred Reporting Items for Systematic Reviews and Meta-Analyses: the PRISMA statement. PLOS Medicine. 2009:DOI: 10.1371/journal.pmed.1000097. PMC3090117 21603045

[35] Kingsland M, Wiggers J, Wolfenden L. Interventions in sports settings to reduce alcohol consumption and alcohol-related harm: a systematic review protocol. BMJ Open. 2012;2(2). doi:10.1136/bmjopen-2011-000645. 10.1136/bmjopen-2011-000645 PMC3329609 22492431

[36] Mercer SL, DeVinney BJ, Fine LJ, Green LW, Dougherty D (2007). Study designs for effectiveness and translation research: identifying trade-offs. Am J Prev Med.

[37] Craig P, Dieppe P, Macintyre S, Michie S, Nazareth I, Petticrew M. Developing and evaluating complex interventions: the new Medical Research Council guidance. BMJ. 2008;337(a1655):doi: http://dx.doi.org/10.1136/bmj.a1655. 10.1136/bmj.a1655 PMC2769032 18824488

[38] Battaglia M, Link M, Frankel M, Osborn L, Mokdad A (2008). An evaluation of respondent selection methods for household mail surveys. Public Opin Q.

[39] Oldendick R, Bishop G, Sorenson S, Tuchfarber A (1998). A comparison of the Kish and last birthday methods of respondent selection in telephone surveys. J Off Stat.

[40] Nutbeam D, Bauman AE (2006). Evaluation in a nutshell: a practical guide to the evaluation of health promotion program.

[41] Sadish W, Cook T, Campbell D (2002). Experimental and quasi-experimental designs for generalized causal inference.

[42] Carr CM (1992). Substance abuse education with elite athletes.

[43] O'Farrell AM (2010). Alcohol intervention programme in a sporting setting: a cluster randomised trial to evaluate a setting-based alcohol intervention programme in the GAA [Thesis submitted for the degree Doctor in Philosophy]: University of Dublin.

[44] Kingsland M, Wolfenden L, Tindall J, Rowland BC, Lecathelinais C, Gillham KE (2015). Tackling risky alcohol consumption in sport: a cluster randomised controlled trial of an alcohol management intervention with community football clubs. J Epidemiol Commun H.

[45] Foxcroft D, Coombes L, Wood S, Allen D, Almeida Santimano N (2014). Motivational interviewing for alcohol misuse in young adults. Cochrane Database Syst Rev.

[46] Foxcroft D, Moreira M, Almeida Santimano N, Smith L (2015). Social norms information for alcohol misuse in university and college students. Cochrane Database Syst Rev.

[47] Durlak J, DuPre E (2008). Implementation matters: a review of research on the influence of implementation on program outcomes and the factors affecting implementation. Am J Community Psychol.

[48] Drygas W, Ruszkowska J, Philpott M, BjÖrkstrÖm O, Parker M, Ireland R (2013). Good practices and health policy analysis in European sports stadia: results from the ‘Healthy Stadia’ project. Health Promot Int.

[49] Lenk KM, Toomey TL, Erickson DJ, Kilian GR, Nelson TF, Fabian LEA (2010). Alcohol control policies and practices at professional sports stadiums. Public Health Rep.

[50] Lyne M, Galloway A (2012). Implementation of effective alcohol control strategies is needed at large sports and entertainment events. Aust N Z J Public Health.

[51] McDonald B, Sylvester K (2014). Learning to get drunk: the importance of drinking in Japanese university sports clubs. Int Rev Sociol Sport.

[52] Bagnardi V, Sorini E, Disalvatore D, Assi V, Corrao G, De Stefani R (2011). ‘Alcohol, less is better’ project: outcomes of an Italian community-based prevention programme on reducing per-capita alcohol consumption. Addiction.

[53] Blaszczynski A (2011). Harm minimization can be achieved by a symbiosis between government, industry and individuals. Addiction.

[54] Bormann CA, Stone MH (2001). The effects of eliminating alcohol in a college stadium: the Folsom Field beer ban. J Am Coll Health.

[55] Caetano R, Pinsky I, Laranjeira R (2012). Should soccer and alcohol mix? Alcohol sales during the 2014 World Soccer Cup games in Brazil. Addiction.

[56] Casswell S, Gilmore L (1989). An evaluated community action project on alcohol. J Stud Alcohol Drugs.

[57] Clarkson JP, Giles-Corti B, Donovan RJ, Frizzell SK (2002). Play hard drink safe: a pilot project to promote responsible alcohol consumption in sporting clubs in Western Australia. Health Promot J Aust.

[58] Donohue B, Pitts M, Gavrilova Y, Ayarza A, Cintron KI (2013). A culturally sensitive approach to treating substance abuse in athletes using evidence-supported methods. J Clinic Sport Psychol.

[59] Duff C, Munro G (2007). Preventing alcohol-related problems in community sports clubs: the good sports program. Subst Use Misuse.

[60] Finch CF, Donaldson A (2010). A sports setting matrix for understanding the implementation context for community sport. Brit J Sport Med.

[61] Fromme K, Marlatt GA, Baer JS, Kivlahan DR (1994). The alcohol skills training program: a group intervention for young adult drinkers. J Subst Abuse Treat.

[62] Gregory BM. College alcohol and life skills study with student-athletes. Dissertation Abstracts International Section A: Humanities and Social Sciences. 2001;62(1-A):89.

[63] Holder HD, Wagenaar AC (1994). Mandated server training and reduced alcohol-involved traffic crashes: a time series analysis of the Oregon experience. Accid Anal Prev.

[64] Kelly L (2011). ‘Social inclusion’through sports-based interventions?. Crit Soc Policy.

[65] Kingsland M, Wolfenden L, Tindall J, Rowland B, Sidey M, McElduff P (2015). Improving the implementation of responsible alcohol management practices by community sporting clubs: a randomised controlled trial. Drug Alcohol Rev.

[66] Maclean A, Bonington J (2008). Legal and regulatory updates sports sponsorship in the UK: the impact of regulatory intervention. J Sponsorship.

[67] Mann RE, Wickens CM (2012). Achieving international progress on alcohol and traffic safety. Addiction.

[68] Marcello R, Danish S, Stolberg A (1989). An evaluation of strategies developed to prevent substance abuse among student-athletes. Sports Pyschol.

[69] Mentha R, Wakerman J (2009). An evaluation of the Australian Football League Central Australian Responsible Alcohol Strategy 2005–07. Health Promot J Austr.

[70] Pridemore WA, Chamlin MB, Kaylen MT, Andreev E (2013). The impact of a national alcohol policy on deaths due to transport accidents in Russia. Addiction.

[71] Reboussin BA, Song EY, Wolfson M (2012). Social influences on the clustering of underage risky drinking and its consequences in communities. J Stud Alcohol Drugs.

[72] Rooney JF (1984). Sports and clean living: a useful myth?. Drug Alcohol Depend.

[73] Rossow I, Norström T (2012). The impact of small changes in bar closing hours on violence. The Norwegian experience from 18 cities. Addiction.

[74] Rowland B, Allen F, Toumbourou JW (2012). Association of risky alcohol consumption and accreditation in the ‘Good Sports’ alcohol management programme. J Epidemiol Commun H.

[75] Schewe G, Knoss HP, Ludwig O, Schaufele A, Schuster R (1984). Experimental studies on the question of the threshold value of alcohol-induced loss of ability in bicycle riding. Blutalkohol.

[76] Shakeshaft A, Doran C, Petrie D, Breen C, Havard A, Abudeen A (2014). The effectiveness of community action in reducing risky alcohol consumption and harm: a cluster randomised controlled trial. PLoS Med.

[77] Stuart GL, Shorey RC, Moore TM, Ramsey SE, Kahler CW, O'Farrell TJ (2013). Randomized clinical trial examining the incremental efficacy of a 90-minute motivational alcohol intervention as an adjunct to standard batterer intervention for men. Addiction.

[78] Thombs D (2000). A test of the perceived norms model to explain drinking patterns among university student athletes. J Am Coll Health.

[79] Tricker R (1996). Drug education and the college athlete: evaluation of a decision-making model. J Drug Educat.

[80] Trolldal B, Brännström L, Paschall MJ, Leifman H (2013). Effects of a multi-component responsible beverage service programme on violent assaults in Sweden. Addiction.

[81] Wagenaar AC, Murray DM, Toomey TL (2000). Communities mobilizing for change on alcohol (CMCA): effects of a randomized trial on arrests and traffic crashes. Addiction.

[82] Warpenius K, Holmila M, Mustonen H (2010). Effects of a community intervention to reduce the serving of alcohol to intoxicated patrons. Addiction.

[83] Watten R (1995). Sports, physical exercise and use of alcohol. Scandinavian J Med Sci Sports.

[84] Yörük BK (2014). Legalization of Sunday alcohol sales and alcohol consumption in the United States. Addiction.

