# Supplementary material for: Interventions in sports settings to reduce risky alcohol consumption and alcohol-related harm: a systematic review
Source: Syst Rev. 2016 Jan 21;5:12. doi: 10.1186/s13643-016-0183-y (PMC4721008; doi:10.1186/s13643-016-0183-y)
Supplement: Additional file 1: — Results of database search. This file contains the search strategy and results of the search executed across the databases described in the methods section. The search was undertaken at two time points to ensure all relevant studies were included. (DOCX 200 kb) [file 13643_2016_183_MOESM1_ESM.docx]

**PART A: Initial database search:**

| **Database** | **Pre-Duplication Results** | **After De-Duplication (using “Find Duplicates”) Results** |
| --- | --- | --- |
| CENTRAL | 140 | 97 |
| CINAHL | 47 | 29 |
| Cochrane Library - Reviews | 3 | 3 |
| Dissertations & Theses | 72 | 68 |
| Embase | 290 | 194 |
| ERIC | 15 | 10 |
| Medline | 242 | 239 |
| Medline In Process | 16 | 13 |
| PsycEXTRA | 4 | 4 |
| PsycINFO | 80 | 44 |
| SportDiscus | 44 | 31 |

**Total results after de-duplication with “Find Duplicates”: 732**

**Symbols used in this document:**

| .mp | Keyword search in OVID databases. This is the broadest search possible |
| --- | --- |
| adj | Adjacency search in OVID databases – “adj3” will retrieve the nominated words within 3 words of each other, in any order |
| / | A slash appearing after a search word/phrase in OVID databases indicates search within the subject heading field |
| * | Truncation symbol – will retrieve all words beginning with the set of letters appearing before the symbol |
| exp | Exploded search in OVID Databases. |
| MH | In EBSCO databases, indicates a search within the subject heading field |
| n | Adjacency search in EBSCO databases – “n3” will retrieve the nominated words within 3 words of each other, in any order. |
| Near/ | Adjacency search in WIley databases – “near/3” will retrieve the nominated words within 3 words of each other, in any order. |
|  |  |

Database: MEDLINE

Name of Host: OVID

Number of results:  **242**

Date searched: 2^nd^ May 2014

Saved Search in DB: Melanie Kingsland – Alcohol and Sports - Medline

| **Set #** | **Search String** | **Results** |
| --- | --- | --- |
| 1 | sport*.mp. or exp Sports/ | 151106 |
| 2 | cricket .*mp | 2204 |
| 3 | netball *.mp | 101 |
| 4 | rugby.mp | 1458 |
| 5 | canoe*.mp | 294 |
| 6 | softball*.mp | 267 |
| 7 | triathl*.mp | 956 |
| 8 | (waterpolo or water polo).mp | 145 |
| 9 | (waterski* or water ski*).mp | 155 |
| 10 | australian rules football*.mp | 139 |
| 11 | surfing.mp | 475 |
| 12 | handball*.mp | 489 |
| 13 | yacht*.mp | 837 |
| 14 | rowing.mp |  |
| 15 | boating.mp | 214 |
| 16 | sailing.mp | 383 |
| 17 | lawn bowls.mp | 4 |
| 18 | bowling.mp | 275 |
| 19 | ((horse or harness or dog or motor or auto or car) adj rac*).mp | 341 |
| 20 | ((motor or auto) adj sport*).mp | 30 |
| 21 | (motorcycl* or motor cycl*).mp | 3183 |
| 22 | archery.mp | 84 |
| 23 | equestrian.mp | 204 |
| 24 | shooting.mp | 2128 |
| 25 | hunting.mp | 3203 |
| 26 | lacrosse.mp | 188 |
| 27 | polo.mp | 1979 |
| 28 | table tennis.mp | 105 |
| 29 | badminton.mp | 190 |
| 30 | squash.mp | 1153 |
| 31 | cycling.mp | 32062 |
| 32 | Fitness Centers/ | 333 |
| 33 | fitness centre*.mp | 42 |
| 34 | gym*.mp | 7144 |
| 35 | (sport* and (game* or event* or club* or arena* or field* or ground*)).mp | 8407 |
| 36 | athlet*.mp | 49402 |
| 37 | player*.mp | 25437 |
| 38 | spectator*.mp | 512 |
| 39 | fan*.mp | 22307 |
| 40 | (sport* and member*).mp | 1125 |
| 41 | 1 or 2 or 3 or 4 or 5 or 6 or 7 or 8 or 9 or 10 or 11 or 12 or 13 or 14 or 15 or 16 or 17 or 18 or 19 or 20 or 21 or 22 or 23 or 24 or 25 or 26 or 27 or 28 or 29 or 30 or 31 or 32 or 33 or 34 or 35 or 36 or 37 or 38 or 39 or 40 | 248010 |
| 42 | exp Health Promotion/ | 53598 |
| 43 | exp Public Health / | 5543957 |
| 44 | Harm Reduction/ | 1562 |
| 45 | (harm* adj3 minimi*).mp | 666 |
| 46 | Health Policy/ | 50110 |
| 47 | Public Policy/ | 28612 |
| 48 | program*.mp | 621997 |
| 49 | intervention*.mp | 523070 |
| 50 | Preventive Medicine/ | 10460 |
| 51 | Health Education/ | 51875 |
| 52 | Consumer Health Information/ | 1728 |
| 53 | Patient Education as Topic/ | 69765 |
| 54 | environment*.mp | 695893 |
| 55 | (responsible and (alcohol* or beverage*) and servic*).mp | 229 |
| 56 | server training.mp | 25 |
| 57 | server intervention*.mp | 17 |
| 58 | enforcement.mp | 8705 |
| 59 | community action*.mp | 540 |
| 60 | community mobili*.mp | 515 |
| 61 | (alcohol* and control*).mp | 58440 |
| 62 | strateg*.mp | 521813 |
| 63 | 42 or 43 or 44 or 45 or 46 or 47 or 48 or 49 or 50 or 51 or 52 or 53 or 54 or 55 or 56 or 57 or 58 or 59 or 60 or 61 or 62 | 6715001 |
| 64 | exp Alcohol Drinking/ | 51180 |
| 65 | alcohol*.mp | 287282 |
| 66 | (alcohol* and (drunk* or incident* or safety or offen* or abus* or disorder* or harm* or violen* or injur* or intoxicat* or assault*)).mp | 95728 |
| 67 | ((drunk* or drink*) and driv*).mp | 4501 |
| 68 | 64 or 65 or 66 or 67 | 288080 |
| 69 | randomized controlled trial.pt | 371440 |
| 70 | controlled clinical trial.pt | 88192 |
| 71 | randomized.ab | 270177 |
| 72 | randomised.ab | 54154 |
| 73 | clinical trials as topic.sh | 169553 |
| 74 | clinical trials.ab | 192124 |
| 75 | trial.tw | 116595 |
| 76 | double blind.tw | 104387 |
| 77 | single blind.tw | 8700 |
| 78 | experiment*.tw | 1256427 |
| 79 | (pretest or pre test).tw | 9951 |
| 80 | (posttest or post test).tw | 10130 |
| 81 | (pre post or prepost).tw | 3928 |
| 82 | before after.tw | 2518 |
| 83 | (quasi randomised or quasi randomized or quazi randomised or quazi randomized).tw | 2575 |
| 84 | stepped wedge.tw | 79 |
| 85 | preference trial.tw | 44 |
| 86 | natural experiment.tw | 703 |
| 87 | ((quasi or quazi) adj experiment*).tw | 5056 |
| 88 | (staggered enrolment trial* or staggered enrollment trial*).tw | 0 |
| 89 | (non randomized or non randomised or nonrandomized or nonrandomised).tw | 13494 |
| 90 | interrupted time series.tw | 924 |
| 91 | (time series and trial).tw | 379 |
| 92 | multiple baseline.tw | 1282 |
| 93 | regression discontinuity.tw | 46 |
| 94 | comprehensive cohort.tw | 46 |
| 95 | 69 or 70 or 71 or 72 or 73 or 74 or 75 or 76 or 77 or 78 or 79 or 80 or 81 or 82 or 83 or 84 or 85 or 86 or 87 or 88 or 89 or 90 or 91 or 92 or 93 or 94 | 2067286 |
| 96 | 41 and 63 and 68 and 95 | 278 |
| **97** | **limit 96 to humans** | **242** |

Database: EMBASE

Name of Host: OVID

Number of results:  **290**

Date searched: 2^nd^ May 2014

Saved Search in DB: Melanie Kingsland – Alcohol and Sports - Embase

| **Set #** | **Search String** | **Results** |
| --- | --- | --- |
| 1 | sport*.mp. or exp Sports/ | 121650 |
| 2 | cricket*.mp | 2108 |
| 3 | netball*.mp | 134 |
| 4 | rugby.mp | 1986 |
| 5 | canoe*.mp | 327 |
| 6 | softball*.mp | 319 |
| 7 | triathl*.mp | 1258 |
| 8 | (waterpolo or water polo).mp | 233 |
| 9 | (waterski* or water ski*).mp | 170 |
| 10 | australian rules football*.mp | 155 |
| 11 | surfing.mp | 665 |
| 12 | handball*.mp | 594 |
| 13 | yacht*.mp | 113 |
| 14 | rowing.mp | 1146 |
| 15 | boating.mp | 280 |
| 16 | sailing.mp | 482 |
| 17 | lawn bowls.mp | 4 |
| 18 | bowling.mp | 358 |
| 19 | ((horse or harness or dog or motor or auto or car) adj rac*).mp | 736 |
| 20 | ((motor or auto) adj sport*).mp | 60 |
| 21 | (motorcycl* or motor cycl*).mp | 3175 |
| 22 | archery.mp | 103 |
| 23 | equestrian.mp | 251 |
| 24 | shooting.mp | 2748 |
| 25 | hunting.mp | 3975 |
| 26 | lacrosse.mp | 223 |
| 27 | polo.mp | 3047 |
| 28 | table tennis.mp | 159 |
| 29 | badminton.mp | 236 |
| 30 | squash.mp | 1780 |
| 31 | cycling.mp | 38977 |
| 32 | Fitness Centers/ | 19788 |
| 33 | fitness centre*.mp | 72 |
| 34 | gym*.mp | 7554 |
| 35 | (sport* and (game* or event* or club* or arena* or field* or ground*)).mp | 13377 |
| 36 | athlet*.mp | 47942 |
| 37 | player*.mp | 33205 |
| 38 | spectator*.mp | 648 |
| 39 | fan*.mp | 25708 |
| 40 | (sport* and member*).mp | 1585 |
| 41 | 1 or 2 or 3 or 4 or 5 or 6 or 7 or 8 or 9 or 10 or 11 or 12 or 13 or 14 or 15 or 16 or 17 or 18 or 19 or 20 or 21 or 22 or 23 or 24 or 25 or 26 or 27 or 28 or 29 or 30 or 31 or 32 or 33 or 34 or 35 or 36 or 37 or 38 or 39 or 40 | 258829 |
| 42 | exp Health Promotion/ | 66091 |
| 43 | exp Public Health/ | 93680 |
| 44 | Harm Reduction/ | 2639 |
| 45 | (harm* adj3 minimi*).mp | 1183 |
| 46 | Health Policy/ | 137777 |
| 47 | Public Policy/ | 63224 |
| 48 | program*.mp | 801864 |
| 49 | intervention*.mp | 706517 |
| 50 | Preventive Medicine/ | 15329 |
| 51 | Health Education/ | 58890 |
| 52 | Consumer Health Information/ | 2221 |
| 53 | Patient Education as Topic/ | 78155 |
| 54 | environment*.mp | 790778 |
| 55 | (responsible and (alcohol* or beverage*) and servic*).mp | 309 |
| 56 | server training.mp | 26 |
| 57 | server intervention*.mp | 19 |
| 58 | enforcement.mp | 13176 |
| 59 | community action*.mp | 552 |
| 60 | community mobili*.mp | 662 |
| 61 | (alcohol* and control*).mp | 138506 |
| 62 | strateg*.mp | 695782 |
| 63 | 42 or 43 or 44 or 45 or 46 or 47 or 48 or 49 or 50 or 51 or 52 or 53 or 54 or 55 or 56 or 57 or 58 or 59 or 60 or 61 or 62 | 3038786 |
| 64 | exp Alcohol Drinking/ | 29680 |
| 65 | alcohol*.mp | 364259 |
| 66 | (alcohol* and (drunk* or incident* or safety or offen* or abus* or disorder* or harm* or violen* or injur* or intoxicat* or assault*)).mp | 123048 |
| 67 | ((drunk* or drink*) and driv*).mp | 5267 |
| 68 | 64 or 65 or 66 or 67 | 371298 |
| 69 | randomized controlled trial/ | 325475 |
| 70 | controlled clinical trial/ | 383189 |
| 71 | randomized.ab | 365158 |
| 72 | randomised.ab | 72197 |
| 73 | "clinical trial (topic)"/ | 40014 |
| 74 | randomly.ab | 245614 |
| 75 | trial.tw | 137574 |
| 76 | double blind.tw | 112590 |
| 77 | single blind.tw | 9998 |
| 78 | experiment*.tw | 1269953 |
| 79 | (pretest or pre test).tw | 13061 |
| 80 | (posttest or post test).tw | 13340 |
| 81 | (pre post or prepost).tw | 6984 |
| 82 | before after.tw | 3681 |
| 83 | (quasi randomised or quasi randomized or quazi randomised or quazi randomized).tw | 2721 |
| 84 | stepped wedge.tw | 119 |
| 85 | preference trial.tw | 58 |
| 86 | natural experiment.tw | 803 |
| 87 | ((quasi or quazi) adj experiment*).tw | 6494 |
| 88 | (staggered enrolment trial* or staggered enrollment trial*).tw | 3 |
| 89 | (non randomized or non randomised or nonrandomized or nonrandomised).tw | 18264 |
| 90 | interrupted time series.tw | 1119 |
| 91 | (time series and trial).tw | 447 |
| 92 | multiple baseline.tw | 1341 |
| 93 | regression discontinuity.tw | 61 |
| 94 | comprehensive cohort.tw | 70 |
| 95 | 69 or 70 or 71 or 72 or 73 or 74 or 75 or 76 or 77 or 78 or 79 or 80 or 81 or 82 or 83 or 84 or 85 or 86 or 87 or 88 or 89 or 90 or 91 or 92 or 93 or 94 | 2172723 |
| 96 | 41 and 63 and 68 and 95 | 410 |
| **97** | **limit 96 to humans** | 290 |

Database: MEDLINE In process

Name of Host: OVID

Number of results:  **16**

Date searched: 2^nd^ May 2014

Saved Search in DB: Melanie Kingsland – Alcohol and Sports – Medline In process

| **Set #** | **Search String** | **Results** |
| --- | --- | --- |
| 1 | sport*.mp | 4921 |
| 2 | cricket*.mp | 225 |
| 3 | netball*.mp | 14 |
| 4 | rugby.mp | 167 |
| 5 | canoe*.mp | 36 |
| 6 | softball*.mp | 31 |
| 7 | triathl*.mp | 102 |
| 8 | (waterpolo or water polo).mp | 20 |
| 9 | (waterski* or water ski*).mp | 14 |
| 10 | australian rules football*.mp | 20 |
| 11 | surfing.mp | 48 |
| 12 | handball*.mp | 72 |
| 13 | yacht*.mp | 9 |
| 14 | rowing.mp | 79 |
| 15 | boating.mp | 22 |
| 16 | sailing.mp | 43 |
| 17 | lawn bowls.mp | 1 |
| 18 | bowling.mp | 32 |
| 19 | ((horse or harness or dog or motor or auto or car) adj rac*).mp | 31 |
| 20 | ((motor or auto) adj sport*).mp | 1 |
| 21 | (motorcycl* or motor cycl*).mp | 229 |
| 22 | archery.mp | 6 |
| 23 | equestrian.mp | 21 |
| 24 | shooting.mp | 285 |
| 25 | hunting.mp | 344 |
| 26 | lacrosse.mp | 26 |
| 27 | polo.mp | 130 |
| 28 | table tennis.mp | 19 |
| 29 | badminton.mp | 21 |
| 30 | squash.mp | 202 |
| 31 | cycling.mp | 3816 |
| 32 | Fitness Centers/ | 0 |
| 33 | fitness centre*.mp | 9 |
| 34 | gym*.mp | 616 |
| 35 | (sport* and (game* or event* or club* or arena* or field* or ground*)).mp | 898 |
| 36 | athlet*.mp | 3785 |
| 37 | player*.mp | 3517 |
| 38 | spectator*.mp | 227 |
| 39 | fan*.mp | 1998 |
| 40 | (sport* and member*).mp | 126 |
| 41 | 1 or 2 or 3 or 4 or 5 or 6 or 7 or 8 or 9 or 10 or 11 or 12 or 13 or 14 or 15 or 16 or 17 or 18 or 19 or 20 or 21 or 22 or 23 or 24 or 25 or 26 or 27 or 28 or 29 or 30 or 31 or 32 or 33 or 34 or 35 or 36 or 37 or 38 or 39 or 40 | 17609 |
| 42 | exp Health Promotion/ | 1633 |
| 43 | exp Public Health/ | 22 |
| 44 | Harm Reduction/ | 1 |
| 45 | (harm* adj3 minimi*).mp | 91 |
| 46 | Health Policy.mp | 1161 |
| 47 | Public Policy.mp | 491 |
| 48 | program*.mp | 45876 |
| 49 | intervention*.mp | 48688 |
| 50 | Preventive Medicine.mp | 602 |
| 51 | Health Education/ | 1644 |
| 52 | Consumer Health Information.mp | 34 |
| 53 | Patient Education.mp | 859 |
| 54 | environment*.mp | 63249 |
| 55 | (responsible and (alcohol* or beverage*) and servic*).mp | 12 |
| 56 | server training.mp | 0 |
| 57 | server intervention*.mp | 2 |
| 58 | enforcement.mp | 672 |
| 59 | community action*.mp | 30 |
| 60 | community mobili*.mp | 77 |
| 61 | (alcohol* and control*).mp | 3326 |
| 62 | strateg*.mp | 59498 |
| 63 | 42 or 43 or 44 or 45 or 46 or 47 or 48 or 49 or 50 or 51 or 52 or 53 or 54 or 55 or 56 or 57 or 58 or 59 or 60 or 61 or 62 | 197192 |
| 64 | exp Alcohol Drinking/ |  |
| 65 | alcohol*.mp | 19712 |
| 66 | (alcohol* and (drunk* or incident* or safety or offen* or abus* or disorder* or harm* or violen* or injur* or intoxicat* or assault*)).mp | 4158 |
| 67 | ((drunk* or drink*) and driv*).mp | 244 |
| 68 | 64 or 65 or 66 or 67 | 19805 |
| 69 | randomized controlled trial.mp | 489 |
| 70 | controlled clinical trial.mp | 49 |
| 71 | randomized.ab | 21579 |
| 72 | randomised.ab | 4333 |
| 73 | clinical trial.mp | 1 |
| 74 | randomly.ab | 19379 |
| 75 | trial.tw | 8879 |
| 76 | double blind.tw | 4776 |
| 77 | single blind.tw | 527 |
| 78 | experiment*.tw | 172596 |
| 79 | (pretest or pre test).tw | 990 |
| 80 | (posttest or post test).tw | 1118 |
| 81 | (pre post or prepost).tw | 635 |
| 82 | before after.tw | 259 |
| 83 | (quasi randomised or quasi randomized or quazi randomised or quazi randomized).tw | 147 |
| 84 | stepped wedge.tw | 28 |
| 85 | preference trial.tw | 2 |
| 86 | natural experiment.tw | 72 |
| 87 | ((quasi or quazi) adj experiment*).tw | 701 |
| 88 | (staggered enrolment trial* or staggered enrollment trial*).tw | 1 |
| 89 | (non randomized or non randomised or nonrandomized or nonrandomised).tw | 1158 |
| 90 | interrupted time series.tw | 110 |
| 91 | (time series and trial).tw | 43 |
| 92 | multiple baseline.tw | 160 |
| 93 | regression discontinuity.tw | 9 |
| 94 | comprehensive cohort.tw | 7 |
| 95 | 69 or 70 or 71 or 72 or 73 or 74 or 75 or 76 or 77 or 78 or 79 or 80 or 81 or 82 or 83 or 84 or 85 or 86 or 87 or 88 or 89 or 90 or 91 or 92 or 93 or 94 | 216630 |
| 96 | 41 and 63 and 68 and 95 | 16 |

Limit to Humans not available in this database

Database: PsycINFO

Name of Host: OVID

Number of results:  **80**

Date searched: 2^nd^ May 2014

Saved Search in DB: Melanie Kingsland – Alcohol and Sports – PsycINFO

| **Set #** | **Search String** | **Results** |
| --- | --- | --- |
| 1 | sport*.mp | 27621 |
| 2 | cricket*.mp | 948 |
| 3 | netball*.mp | 75 |
| 4 | rugby.mp | 398 |
| 5 | canoe*.mp | 146 |
| 6 | softball*.mp | 251 |
| 7 | triathl*.mp | 131 |
| 8 | (waterpolo or water polo).mp | 38 |
| 9 | (waterski* or water ski*).mp | 19 |
| 10 | australian rules football*.mp | 35 |
| 11 | surfing.mp | 308 |
| 12 | handball*.mp | 226 |
| 13 | yacht*.mp | 30 |
| 14 | rowing.mp | 142 |
| 15 | boating.mp | 67 |
| 16 | sailing.mp | 224 |
| 17 | lawn bowls.mp | 4 |
| 18 | bowling.mp | 329 |
| 19 | ((horse or harness or dog or motor or auto or car) adj rac*).mp | 277 |
| 20 | ((motor or auto) adj sport*).mp | 20 |
| 21 | (motorcycl* or motor cycl*).mp | 669 |
| 22 | archery.mp | 83 |
| 23 | equestrian.mp | 59 |
| 24 | shooting.mp | 1550 |
| 25 | hunting.mp | 1672 |
| 26 | lacrosse.mp | 74 |
| 27 | polo.mp | 97 |
| 28 | table tennis.mp | 148 |
| 29 | badminton.mp | 120 |
| 30 | squash.mp | 74 |
| 31 | cycling.mp | 3172 |
| 32 | Fitness Centers/ | 0 |
| 33 | fitness centre*.mp | 27 |
| 34 | gym*.mp | 1877 |
| 35 | (sport* and (game* or event* or club* or arena* or field* or ground*)).mp | 7092 |
| 36 | athlet*.mp | 18459 |
| 37 | player*.mp | 12099 |
| 38 | spectator*.mp | 1606 |
| 39 | fan*.mp | 20607 |
| 40 | (sport* and member*).mp | 1294 |
| 41 | 1 or 2 or 3 or 4 or 5 or 6 or 7 or 8 or 9 or 10 or 11 or 12 or 13 or 14 or 15 or 16 or 17 or 18 or 19 or 20 or 21 or 22 or 23 or 24 or 25 or 26 or 27 or 28 or 29 or 30 or 31 or 32 or 33 or 34 or 35 or 36 or 37 or 38 or 39 or 40 | 71299 |
| 42 | exp Health Promotion/ | 14842 |
| 43 | exp Public Health/ | 13769 |
| 44 | Harm Reduction/ | 1753 |
| 45 | (harm* adj3 minimi*).mp | 604 |
| 46 | Health Policy.mp | 4390 |
| 47 | Public Policy.mp | 15594 |
| 48 | program*.mp | 308292 |
| 49 | intervention*.mp | 238932 |
| 50 | Preventive Medicine.mp | 2119 |
| 51 | Health Education/ | 9677 |
| 52 | Consumer Health Information.mp | 39 |
| 53 | Patient Education.mp | 2266 |
| 54 | environment*.mp | 270543 |
| 55 | (responsible and (alcohol* or beverage*) and servic*).mp | 163 |
| 56 | server training.mp | 18 |
| 57 | server intervention*.mp | 27 |
| 58 | enforcement.mp | 8423 |
| 59 | community action*.mp | 564 |
| 60 | community mobili*.mp | 325 |
| 61 | (alcohol* and control*).mp | 21200 |
| 62 | strateg*.mp | 230787 |
| 63 | 42 or 43 or 44 or 45 or 46 or 47 or 48 or 49 or 50 or 51 or 52 or 53 or 54 or 55 or 56 or 57 or 58 or 59 or 60 or 61 or 62 | 907093 |
| 64 | exp Alcohol Drinking/ | 0 |
| 65 | alcohol*.mp | 103309 |
| 66 | (alcohol* and (drunk* or incident* or safety or offen* or abus* or disorder* or harm* or violen* or injur* or intoxicat* or assault*)).mp | 62808 |
| 67 | ((drunk* or drink*) and driv*).mp | 3305 |
| 68 | 64 or 65 or 66 or 67 | 104090 |
| 69 | randomized controlled trial.mp |  |
| 70 | controlled clinical trial.mp |  |
| 71 | randomized.ab | 37989 |
| 72 | randomised.ab | 4582 |
| 73 | clinical trial.mp | 14783 |
| 74 | randomly.ab | 50426 |
| 75 | trial.tw | 17207 |
| 76 | double blind.tw | 17784 |
| 77 | single blind.tw | 1250 |
| 78 | experiment*.tw | 321498 |
| 79 | (pretest or pre test).tw | 12397 |
| 80 | (posttest or post test).tw | 16566 |
| 81 | (pre post or prepost).tw | 3744 |
| 82 | before after.tw | 1187 |
| 83 | (quasi randomised or quasi randomized or quazi randomised or quazi randomized).tw | 99 |
| 84 | stepped wedge.tw | 10 |
| 85 | preference trial.tw | 21 |
| 86 | natural experiment.tw | 521 |
| 87 | ((quasi or quazi) adj experiment*).tw | 6631 |
| 88 | (staggered enrolment trial* or staggered enrollment trial*).tw | 0 |
| 89 | (non randomized or non randomised or nonrandomized or nonrandomised).tw | 1234 |
| 90 | interrupted time series.tw | 444 |
| 91 | (time series and trial).tw | 100 |
| 92 | multiple baseline.tw | 3754 |
| 93 | regression discontinuity.tw | 158 |
| 94 | comprehensive cohort.tw | 7 |
| 95 | 69 or 70 or 71 or 72 or 73 or 74 or 75 or 76 or 77 or 78 or 79 or 80 or 81 or 82 or 83 or 84 or 85 or 86 or 87 or 88 or 89 or 90 or 91 or 92 or 93 or 94 | 427648 |
| 96 | 41 and 63 and 68 and 95 | 80 |
| **97** | **Limit 96 to human** | **76** |

PsycEXTRA - 4

Database: CINAHL

Name of Host: EBSCO

Number of results:  **47**

Date searched: 2^nd^ May 2014

Saved Search in DB: Melanie Kingsland – Alcohol and Sports - CINAHL

| **Set #** | **Search String** | **Results** |
| --- | --- | --- |
| 1 | sport* | 32,212 |
| 2 | cricket | 274 |
| 3 | netball * | 69 |
| 4 | rugby | 1,170 |
| 5 | canoe* | 85 |
| 6 | softball* | 198 |
| 7 | triathl* | 647 |
| 8 | (waterpolo or water polo) | 89 |
| 9 | (waterski* or water ski*) | 247 |
| 10 | australian rules football* | 75 |
| 11 | surfing | 347 |
| 12 | handball* | 258 |
| 13 | yacht* | 31 |
| 14 | rowing | 680 |
| 15 | boating | 41 |
| 16 | sailing | 221 |
| 17 | lawn bowls | 2 |
| 18 | bowling | 223 |
| 19 | ((horse or harness or dog or motor or auto or car) n1 rac*) | 90 |
| 20 | ((motor or auto) n1 sport*) | 221 |
| 21 | (motorcycl* or motor cycl*) | 706 |
| 22 | archery | 38 |
| 23 | equestrian | 40 |
| 24 | shooting | 597 |
| 25 | hunting | 359 |
| 26 | lacrosse | 113 |
| 27 | polo | 142 |
| 28 | table tennis | 43 |
| 29 | badminton | 80 |
| 30 | squash | 215 |
| 31 | cycling | 6,897 |
| 32 | (MH "Fitness Centers") | 997 |
| 33 | fitness centre* | 43 |
| 34 | gym* | 1,480 |
| 35 | (sport* and (game* or event* or club* or arena* or field* or ground*)) | 7,239 |
| 36 | (MH "Athletes") OR "athlet*" | 36,767 |
| 37 | player* | 7,872 |
| 38 | spectator* | 147 |
| 39 | fan* | 2,443 |
| 40 | (sport* and member*) | 584 |
| 41 | 1 or 2 or 3 or 4 or 5 or 6 or 7 or 8 or 9 or 10 or 11 or 12 or 13 or 14 or 15 or 16 or 17 or 18 or 19 or 20 or 21 or 22 or 23 or 24 or 25 or 26 or 27 or 28 or 29 or 30 or 31 or 32 or 33 or 34 or 35 or 36 or 37 or 38 or 39 or 40 | 67,914 |
| 42 | (MH "Health Promotion+") | 37,531 |
| 43 | (MH "Public Health+") | 645,066 |
| 44 | (MH "Harm Reduction") | 1,710 |
| 45 | (harm* n3 minimi*) | 349 |
| 46 | (MH "Health Policy") | 32,315 |
| 47 | (MH "Public Policy") | 13,573 |
| 48 | program* | 271,007 |
| 49 | intervention* | 190,238 |
| 50 | (MH "Preventive Health Care") OR "Preventive Medicine" | 13,470 |
| 51 | (MH "Health Education") | 17,513 |
| 52 | (MH "Consumer Health Information") | 8,861 |
| 53 | (MH "Patient Education") | 46,826 |
| 54 | environment* | 123,698 |
| 55 | (responsible and (alcohol* or beverage*) and servic*) | 86 |
| 56 | server training | 11 |
| 57 | server intervention* | 12 |
| 58 | enforcement | 2,453 |
| 59 | community action* | 831 |
| 60 | community mobili* | 687 |
| 61 | (alcohol* and control*) | 15,428 |
| 62 | strateg* | 121,542 |
| 63 | 42 or 43 or 44 or 45 or 46 or 47 or 48 or 49 or 50 or 51 or 52 or 53 or 54 or 55 or 56 or 57 or 58 or 59 or 60 or 61 or 62 | 1,185,825 |
| 64 | exp Alcohol Drinking/ | 16,427 |
| 65 | alcohol* | 52,159 |
| 66 | (alcohol* and (drunk* or incident* or safety or offen* or abus* or disorder* or harm* or violen* or injur* or intoxicat* or assault*)) | 26,906 |
| 67 | ((drunk* or drink*) and driv*) | 1,183 |
| 68 | 64 or 65 or 66 or 67 | 52,321 |
| 69 | (MH " Randomized Controlled Trials ") | 30,900 |
| 70 | (MH "Clinical Trials") | 113,272 |
| 71 | AB randomized | 53,106 |
| 72 | AB randomised | 14,017 |
| 73 | controlled clinical trial | 5,353 |
| 74 | AB clinical trials | 20,546 |
| 75 | trial | 85,198 |
| 76 | double blind | 33,150 |
| 77 | single blind | 8,988 |
| 78 | experiment* | 64,440 |
| 79 | (pretest or pre test) | 28,627 |
| 80 | (posttest or post test) | 29,566 |
| 81 | (pre post or prepost) | 10,931 |
| 82 | before after | 31,092 |
| 83 | (quasi randomised or quasi randomized or quazi randomised or quazi randomized) | 1,614 |
| 84 | stepped wedge | 42 |
| 85 | preference trial | 60 |
| 86 | natural experiment | 255 |
| 87 | ((quasi or quazi) n1 experiment*) | 7,675 |
| 88 | (staggered enrolment trial* or staggered enrollment trial*) | 0 |
| 89 | (non randomized or non randomised or nonrandomized or nonrandomised) | 3,617 |
| 90 | interrupted time series | 487 |
| 91 | (time series and trial) | 173 |
| 92 | multiple baseline | 816 |
| 93 | regression discontinuity | 26 |
| 94 | comprehensive cohort | 54 |
| 95 | 69 or 70 or 71 or 72 or 73 or 74 or 75 or 76 or 77 or 78 or 79 or 80 or 81 or 82 or 83 or 84 or 85 or 86 or 87 or 88 or 89 or 90 or 91 or 92 or 93 or 94 | 325,880 |
| 96 | 41 and 63 and 68 and 95 | 54 |
| **97** | **limit 96 to humans** | **47** |

Database: SPORTDISCUS

Name of Host: EBSCO

Number of results:  **44**

Date searched: 2^nd^ May 2014

Saved Search in DB: Melanie Kingsland – Alcohol and Sports - SportDiscus

| **Set #** | **Search String** | **Results** |
| --- | --- | --- |
| 1 | sport* | 844,676 |
| 2 | cricket | 8,664 |
| 3 | netball * | 1,304 |
| 4 | rugby | 48,616 |
| 5 | canoe* | 7,764 |
| 6 | softball* | 4,665 |
| 7 | triathl* | 17,038 |
| 8 | (waterpolo or water polo) | 2,341 |
| 9 | (waterski* or water ski*) | 2,367 |
| 10 | australian rules football* | 484 |
| 11 | surfing | 5,407 |
| 12 | handball* | 9,893 |
| 13 | yacht* | 19,137 |
| 14 | rowing | 8,546 |
| 15 | boating | 7,668 |
| 16 | sailing | 6,460 |
| 17 | lawn bowls | 980 |
| 18 | bowling | 10,569 |
| 19 | ((horse or harness or dog or motor or auto or car) n1 rac*) | 13,451 |
| 20 | ((motor or auto) n1 sport*) | 16,830 |
| 21 | (motorcycl* or motor cycl*) | 21,558 |
| 22 | archery | 5,181 |
| 23 | equestrian | 5,487 |
| 24 | shooting | 11,982 |
| 25 | hunting | 8,282 |
| 26 | lacrosse | 6,122 |
| 27 | polo | 3,434 |
| 28 | table tennis | 2,708 |
| 29 | badminton | 3,107 |
| 30 | squash | 2,357 |
| 31 | cycling | 51,424 |
| 32 | Fitness center* |  |
| 33 | fitness centre* | 460 |
| 34 | gym* | 29,920 |
| 35 | (sport* and (game* or event* or club* or arena* or field* or ground*)) | 208,621 |
| 36 | (MH "Athletes") OR "athlet*" | 303,494 |
| 37 | player* | 189,030 |
| 38 | spectator* | 9,588 |
| 39 | fan* | 23,417 |
| 40 | (sport* and member*) | 16,192 |
| 41 | 1 or 2 or 3 or 4 or 5 or 6 or 7 or 8 or 9 or 10 or 11 or 12 or 13 or 14 or 15 or 16 or 17 or 18 or 19 or 20 or 21 or 22 or 23 or 24 or 25 or 26 or 27 or 28 or 29 or 30 or 31 or 32 or 33 or 34 or 35 or 36 or 37 or 38 or 39 or 40 | 1,103,287 |
| 42 | Health Promotion | 13,246 |
| 43 | Public Health | 33,386 |
| 44 | Harm Reduction | 672 |
| 45 | (harm* n3 minimi*) | 166 |
| 46 | Health Policy | 3,827 |
| 47 | Public Policy | 3,071 |
| 48 | program* | 152,484 |
| 49 | intervention* | 34,417 |
| 50 | "Preventive Medicine" | 5,234 |
| 51 | Health Education | 23,606 |
| 52 | Consumer Health Information | 64 |
| 53 | Patient Education | 1,125 |
| 54 | environment* | 43,517 |
| 55 | (responsible and (alcohol* or beverage*) and servic*) | 44 |
| 56 | server training | 15 |
| 57 | server intervention* | 9 |
| 58 | enforcement | 2,274 |
| 59 | community action* | 293 |
| 60 | community mobili* | 233 |
| 61 | (alcohol* and control*) | 2,948 |
| 62 | strateg* | 52,719 |
| 63 | 42 or 43 or 44 or 45 or 46 or 47 or 48 or 49 or 50 or 51 or 52 or 53 or 54 or 55 or 56 or 57 or 58 or 59 or 60 or 61 or 62 | 296,417 |
| 64 | exp Alcohol Drinking/ | 1,294 |
| 65 | alcohol* | 16,701 |
| 66 | (alcohol* and (drunk* or incident* or safety or offen* or abus* or disorder* or harm* or violen* or injur* or intoxicat* or assault*)) | 9,026 |
| 67 | ((drunk* or drink*) and driv*) | 860 |
| 68 | 64 or 65 or 66 or 67 | 17,054 |
| 69 | Randomized Controlled Trials | 6,982 |
| 70 | Clinical Trials | 9,987 |
| 71 | randomized | 11,529 |
| 72 | randomised | 2,567 |
| 73 | controlled clinical trial | 820 |
| 74 | clinical trials | 4,762 |
| 75 | trial | 37,644 |
| 76 | double blind | 4,233 |
| 77 | single blind | 599 |
| 78 | experiment* | 43,750 |
| 79 | (pretest or pre test) | 4,141 |
| 80 | (posttest or post test) | 4,877 |
| 81 | (pre post or prepost) | 6,302 |
| 82 | before after | 13,957 |
| 83 | (quasi randomised or quasi randomized or quazi randomised or quazi randomized) | 86 |
| 84 | stepped wedge | 1 |
| 85 | preference trial | 32 |
| 86 | natural experiment | 103 |
| 87 | ((quasi or quazi) n1 experiment*) | 629 |
| 88 | (staggered enrolment trial* or staggered enrollment trial*) | 3 |
| 89 | (non randomized or non randomised or nonrandomized or nonrandomised) | 564 |
| 90 | interrupted time series | 59 |
| 91 | (time series and trial) | 152 |
| 92 | multiple baseline | 285 |
| 93 | regression discontinuity | 5 |
| 94 | comprehensive cohort | 10 |
| 95 | 69 or 70 or 71 or 72 or 73 or 74 or 75 or 76 or 77 or 78 or 79 or 80 or 81 or 82 or 83 or 84 or 85 or 86 or 87 or 88 or 89 or 90 or 91 or 92 or 93 or 94 | 97,054 |
| 96 | 41 and 63 and 68 and 95 | 44 |

Limit to Humans not available in this database

**COCHRANE LIBRARY**

**Cochrane Reviews – 3**

**CENTRAL – 140**

'(Sport* or cricket* or netball* or rugby* or canoe* or softball* or triathl* or waterpolo or "water polo" or waterski or "water ski*" or "Australian rules football*" or surfing or handball* or yacht* or rowing or boating or sailing or bowl* or "horse rac*" or "harness rac*" or "dog rac*" or "motor rac*" or "auto rac*" or "car rac*" or "motor sport*" or "auto sport*" or archery or equestrian or shooting or hunting or lacrosse or polo or "table tennis" or badminton or squash or cycling or "fitness center*" or "fitness centre*" or gym* or (sport* and (game* or event* or club* or arena* or field* or ground*)) or player* or athlet* or spectator* or fan* or (sport* and member*)) in Title, Abstract, Keywords and ("health promotion" or "public health" or "harm reduction" or "harm minimi*" or "health policy" or "public policy" or program or intervention* or "preventive medicine" or "health education" or "consumer health information" or "patient education" or environment* or (responsible and (alcohol* or beverage*) and servic*) or "server training" or "server intervention*" or enforcement or "community action*" or "community mobili*" or (alcohol* and control*) or strateg*) in Title, Abstract, Keywords and (Alcohol* or "drunk* driv*" or "drink* driv*") in Title, Abstract, Keywords and (Trial* or randomi* or "double blind" or "single blind" or experiment* or pretest or "pre test" or posttest or "post test" or "pre post" or prepost or "before after" or "quasi randomised" or "quasi randomized" or "quazi randomised" or "quazi randomized" or "stepped wedge" or "non randomized" or "non randomised" or nonrandomized or nonrandomised or "time series" or "multiple baseline" or "regression discontinuity" or "comprehensive cohort")

**DISSERTATIONS AND THESES - 72**

**ERIC - 15**

(Sport* or cricket* or netball* or rugby* or canoe* or softball* or triathl* or waterpolo or “water polo” or waterski or “water ski*” or “Australian rules football*” or surfing or handball* or yacht* or rowing or boating or sailing or bowl* or “horse rac*” or “harness rac*” or “dog rac*” or “motor rac*” or “auto rac*” or “car rac*” or “motor sport*” or “auto sport*” or archery or equestrian or shooting or hunting or lacrosse or polo or “table tennis” or badminton or squash or cycling or “fitness center*” or “fitness centre*” or gym* or (sport* and (game* or event* or club* or arena* or field* or ground*)) or player* or athlet* or spectator* or fan* or (sport* and member*))

AND

(“health promotion” or “public health” or “harm reduction” or “harm minimi*” or “health policy” or “public policy” or program or intervention* or “preventive medicine” or “health education” or “consumer health information” or “patient education” or environment* or (responsible and (alcohol* or beverage*) and servic*) or “server training” or “server intervention*” or enforcement or “community action*” or “community mobili*” or (alcohol* and control*) or strateg*)

AND

(Alcohol* or “drunk* driv*” or “drink* driv*”)

AND

(Trial* or randomi* or “double blind” or “single blind” or experiment* or pretest or “pre test” or posttest or “post test” or “pre post” or prepost or “before after” or “quasi randomised” or “quasi randomized” or “quazi randomised” or “quazi randomized” or “stepped wedge” or “non randomized” or “non randomised” or nonrandomized or nonrandomised or “time series” or “multiple baseline” or “regression discontinuity” or “comprehensive cohort”)

**PART B: Re-run of database search to bring up-to-date:**

**Total results before de-duplication= 152**

**Total results after de-duplication with “Find Duplicates”: 145**

**After removing duplicate by hand search: 115**

Database: MEDLINE

Name of Host: OVID

Number of results:  **236. (16 from 2014-15)**

Date searched: 20th August 2015

Shakeshaft??

| 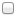 | 1 | sport*.mp. or exp Sports/ | 164131 |
| --- | --- | --- | --- |
| 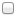 | 2 | cricket*.mp. | 2378 |
| 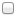 | 3 | netball*.mp. | 115 |
| 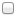 | 4 | rugby.mp. | 1609 |
| 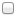 | 5 | canoe*.mp. | 320 |
| 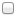 | 6 | softball*.mp. | 288 |
| 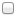 | 7 | triathl*.mp. | 1045 |
| 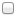 | 8 | (waterpolo or water polo).mp. | 169 |
| 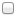 | 9 | (waterski* or water ski*).mp. | 163 |
| 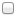 | 10 | australian rules football*.mp. | 153 |
| 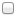 | 11 | surfing.mp. | 526 |
| 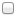 | 12 | handball*.mp. | 556 |
| 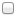 | 13 | yacht*.mp. | 94 |
| 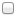 | 14 | rowing.mp. | 901 |
| 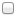 | 15 | boating.mp. | 236 |
| 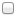 | 16 | sailing.mp. | 412 |
| 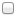 | 17 | lawn bowls.mp. | 5 |
| 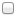 | 18 | bowling.mp. | 293 |
| 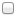 | 19 | ((horse or harness or dog or motor or auto or car) adj rac*).mp. | 362 |
| 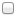 | 20 | ((motor or auto) adj sport*).mp. | 30 |
| 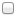 | 21 | (motorcycl* or motor cycl*).mp. | 3432 |
| 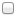 | 22 | archery.mp. | 90 |
| 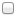 | 23 | equestrian.mp. | 233 |
| 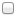 | 24 | shooting.mp. | 2310 |
| 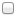 | 25 | hunting.mp. | 3604 |
| 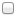 | 26 | lacrosse.mp. | 202 |
| 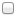 | 27 | polo.mp. | 2266 |
| 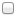 | 28 | table tennis.mp. | 130 |
| 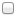 | 29 | badminton.mp. | 206 |
| 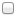 | 30 | squash.mp. | 1194 |
| 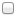 | 31 | cycling.mp. | 34874 |
| 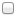 | 32 | Fitness Centers/ | 365 |
| 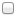 | 33 | fitness centre*.mp. | 49 |
| 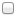 | 34 | gym*.mp. | 7677 |
| 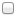 | 35 | (sport* and (game* or event* or club* or arena* or field* or ground*)).mp. | 9305 |
| 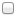 | 36 | athlet*.mp. | 53904 |
| 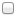 | 37 | player*.mp. | 29329 |
| 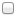 | 38 | spectator*.mp. | 557 |
| 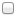 | 39 | fan*.mp. | 23700 |
| 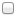 | 40 | (sport* and member*).mp. | 1252 |
| 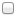 | 41 | 1 or 2 or 3 or 4 or 5 or 6 or 7 or 8 or 9 or 10 or 11 or 12 or 13 or 14 or 15 or 16 or 17 or 18 or 19 or 20 or 21 or 22 or 23 or 24 or 25 or 26 or 27 or 28 or 29 or 30 or 31 or 32 or 33 or 34 or 35 or 36 or 37 or 38 or 39 or 40 | 270267 |
| 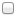 | 42 | exp Health Promotion/ | 59217 |
| 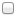 | 43 | exp Public Health/ | 6018280 |
| 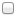 | 44 | Harm Reduction/ | 1814 |
| 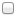 | 45 | (harm* adj3 minimi*).mp. | 757 |
| 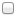 | 46 | Health Policy/ | 54161 |
| 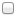 | 47 | Public Policy/ | 28786 |
| 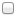 | 48 | program*.mp. | 672109 |
| 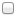 | 49 | intervention*.mp. | 589865 |
| 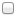 | 50 | Preventive Medicine/ | 10860 |
| 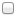 | 51 | Health Education/ | 53927 |
| 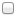 | 52 | Consumer Health Information/ | 2192 |
| 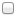 | 53 | Patient Education as Topic/ | 73363 |
| 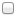 | 54 | environment*.mp. | 765806 |
| 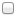 | 55 | (responsible and (alcohol* and beverage*) and servic*).mp. | 56 |
| 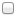 | 56 | server training.mp. | 25 |
| 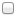 | 57 | enforcement.mp. | 9542 |
| 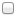 | 58 | community action*.mp. | 568 |
| 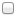 | 59 | community mobili*.mp. | 596 |
| 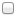 | 60 | (alcohol* and control*).mp. | 63528 |
| 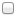 | 61 | strateg*.mp. | 589637 |
| 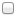 | 62 | server intervention*.mp. | 18 |
| 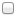 | 63 | 42 or 43 or 44 or 45 or 46 or 47 or 48 or 49 or 50 or 51 or 52 or 53 or 54 or 55 or 56 or 57 or 58 or 59 or 60 or 61 or 62 | 7298465 |
| 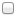 | 64 | exp Alcohol Drinking/ | 55480 |
| 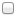 | 65 | alcohol*.mp. | 306315 |
| 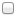 | 66 | (alcohol* and (drunk* or incident* or safety or offen* or abus* or disorder* or harm* or violen* or injur* or intoxicat* or assault*)).mp. | 103201 |
| 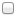 | 67 | ((drunk* or drink*) and driv*).mp. | 4938 |
| 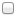 | 68 | 64 or 65 or 66 or 67 | 307247 |
| 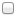 | 69 | randomized controlled trial.pt. | 407796 |
| 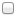 | 70 | controlled clinical trial.pt. | 91378 |
| 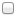 | 71 | randomized.ab. | 300404 |
| 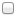 | 72 | randomised.ab. | 61268 |
| 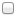 | 73 | clinical trials as topic.sh. | 177916 |
| 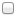 | 74 | clinical trials.ab. | 136565 |
| 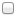 | 75 | trial.tw. | 370746 |
| 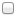 | 76 | double blind.tw. | 110776 |
| 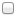 | 77 | single blind.tw. | 9397 |
| 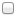 | 78 | experiment*.tw. | 1338691 |
| 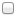 | 79 | (pretest or Pre test).tw. | 10946 |
| 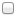 | 80 | (posttest or post test).tw. | 11228 |
| 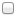 | 81 | (pre post or prepost).tw. | 4692 |
| 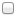 | 82 | before after.tw. | 2895 |
| 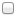 | 83 | (quasi randomised or quasi randomized or quazi randomised or quazi randomized).tw. | 2853 |
| 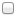 | 84 | stepped wedge.tw. | 128 |
| 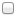 | 85 | preference trial.tw. | 45 |
| 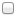 | 86 | natural experiment.tw. | 796 |
| 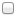 | 87 | ((quasi or quazi) adj experiment*).tw. | 5852 |
| 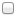 | 88 | (staggered enrolment trial* or staggered enrollment trial*).tw. | 0 |
| 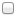 | 89 | (non randomized or non randomised or nonrandomized or nonrandomised).tw. | 15093 |
| 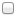 | 90 | interrupted time series.tw. | 1131 |
| 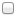 | 91 | (time series and trial).tw. | 428 |
| 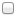 | 92 | multiple baseline.tw. | 1365 |
| 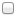 | 93 | regression discontinuity.tw. | 65 |
| 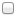 | 94 | comprehensive cohort.tw. | 53 |
| 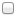 | 95 | 69 or 70 or 71 or 72 or 73 or 74 or 75 or 76 or 77 or 78 or 79 or 80 or 81 or 82 or 83 or 84 or 85 or 86 or 87 or 88 or 89 or 90 or 91 or 92 or 93 or 94 | 2283793 |
| 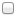 | 96 | 41 and 63 and 68 and 95 | 274 |
| 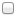 | 97 | limit 96 to humans | 236 |
| 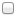 | 98 | limit 97 to yr="2014 -Current" | 16 |

Database: EMBASE

Name of Host: OVID

Number of results:  **385 (58 from 2014-15)**

Date searched: 20^th^ August 2015

Unsure: Edwards?

|  | 1 | sport*.mp. or exp Sports/ | 171987 |
| --- | --- | --- | --- |
| 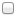 | 2 | cricket*.mp. | 2986 |
| 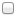 | 3 | netball*.mp. | 171 |
|  | 4 | rugby.mp. | 2543 |
|  | 5 | canoe*.mp. | 467 |
|  | 6 | softball*.mp. | 415 |
|  | 7 | triathl*.mp. | 1503 |
|  | 8 | (waterpolo or water polo).mp. | 305 |
|  | 9 | (waterski* or water ski*).mp. | 263 |
|  | 10 | australian rules football*.mp. | 189 |
|  | 11 | surfing.mp. | 778 |
|  | 12 | handball*.mp. | 797 |
|  | 13 | yacht*.mp. | 153 |
|  | 14 | rowing.mp. | 1547 |
|  | 15 | boating.mp. | 352 |
|  | 16 | sailing.mp. | 666 |
|  | 17 | lawn bowls.mp. | 6 |
|  | 18 | bowling.mp. | 503 |
|  | 19 | ((horse or harness or dog or motor or auto or car) adj rac*).mp. | 494 |
|  | 20 | ((motor or auto) adj sport*).mp. | 72 |
|  | 21 | (motorcycl* or motor cycl*).mp. | 4487 |
|  | 22 | archery.mp. | 161 |
|  | 23 | equestrian.mp. | 319 |
|  | 24 | shooting.mp. | 3721 |
|  | 25 | hunting.mp. | 5169 |
|  | 26 | lacrosse.mp. | 339 |
|  | 27 | polo.mp. | 3779 |
|  | 28 | table tennis.mp. | 243 |
|  | 29 | badminton.mp. | 326 |
|  | 30 | squash.mp. | 2622 |
|  | 31 | cycling.mp. | 49219 |
|  | 32 | Fitness Centers/ | 25939 |
|  | 33 | fitness centre*.mp. | 88 |
|  | 34 | gym*.mp. | 12125 |
|  | 35 | (sport* and (game* or event* or club* or arena* or field* or ground*)).mp. | 17361 |
|  | 36 | athlet*.mp. | 64742 |
|  | 37 | player*.mp. | 42626 |
|  | 38 | spectator*.mp. | 843 |
|  | 39 | fan*.mp. | 39049 |
|  | 40 | (sport* and member*).mp. | 2130 |
|  | 41 | 1 or 2 or 3 or 4 or 5 or 6 or 7 or 8 or 9 or 10 or 11 or 12 or 13 or 14 or 15 or 16 or 17 or 18 or 19 or 20 or 21 or 22 or 23 or 24 or 25 or 26 or 27 or 28 or 29 or 30 or 31 or 32 or 33 or 34 or 35 or 36 or 37 or 38 or 39 or 40 | 357513 |
|  | 42 | exp Health Promotion/ | 75679 |
|  | 43 | exp Public Health/ | 137981 |
|  | 44 | Harm Reduction/ | 3344 |
|  | 45 | (harm* adj3 minimi*).mp. | 1403 |
|  | 46 | Health Policy/ | 154876 |
|  | 47 | Public Policy/ | 82215 |
|  | 48 | program*.mp. | 1052254 |
|  | 49 | intervention*.mp. | 887240 |
|  | 50 | Preventive Medicine/ | 25491 |
|  | 51 | Health Education/ | 84766 |
|  | 52 | Consumer Health Information/ | 2697 |
|  | 53 | Patient Education as Topic/ | 92066 |
|  | 54 | environment*.mp. | 1053887 |
|  | 55 | (responsible and (alcohol* and beverage*) and servic*).mp. | 73 |
|  | 56 | server training.mp. | 26 |
|  | 57 | enforcement.mp. | 16552 |
|  | 58 | community action*.mp. | 785 |
|  | 59 | community mobili*.mp. | 816 |
|  | 60 | (alcohol* and control*).mp. | 170024 |
|  | 61 | strateg*.mp. | 848878 |
|  | 62 | server intervention*.mp. | 21 |
|  | 63 | 42 or 43 or 44 or 45 or 46 or 47 or 48 or 49 or 50 or 51 or 52 or 53 or 54 or 55 or 56 or 57 or 58 or 59 or 60 or 61 or 62 | 3918107 |
|  | 64 | exp Alcohol Drinking/ | 39085 |
|  | 65 | alcohol*.mp. | 540113 |
|  | 66 | (alcohol* and (drunk* or incident* or safety or offen* or abus* or disorder* or harm* or violen* or injur* or intoxicat* or assault*)).mp. | 174355 |
|  | 67 | ((drunk* or drink*) and driv*).mp. | 7216 |
|  | 68 | 64 or 65 or 66 or 67 | 550584 |
|  | 69 | randomized controlled trial/ | 382881 |
|  | 70 | controlled clinical trial/ | 392352 |
|  | 71 | randomised.ab. | 87512 |
|  | 72 | randomized.ab. | 445212 |
|  | 73 | "clinical trial (topic)"/ | 62290 |
|  | 74 | randomly.ab. | 302514 |
|  | 75 | trial.tw. | 553555 |
|  | 76 | double blind.tw. | 153564 |
|  | 77 | single blind.tw. | 12929 |
|  | 78 | experiment*.tw. | 1905604 |
|  | 79 | (pretest or Pre test).tw. | 16515 |
|  | 80 | (posttest or post test).tw. | 16898 |
|  | 81 | (pre post or prepost).tw. | 9240 |
|  | 82 | before after.tw. | 4837 |
|  | 83 | (quasi randomised or quasi randomized or quazi randomised or quazi randomized).tw. | 3101 |
|  | 84 | stepped wedge.tw. | 227 |
|  | 85 | preference trial.tw. | 72 |
|  | 86 | natural experiment.tw. | 1035 |
|  | 87 | ((quasi or quazi) adj experiment*).tw. | 8101 |
|  | 88 | (staggered enrolment trial* or staggered enrollment trial*).tw. | 3 |
|  | 89 | (non randomized or non randomised or nonrandomized or nonrandomised).tw. | 21735 |
|  | 90 | interrupted time series.tw. | 1508 |
|  | 91 | (time series and trial).tw. | 558 |
|  | 92 | multiple baseline.tw. | 1990 |
|  | 93 | regression discontinuity.tw. | 94 |
|  | 94 | comprehensive cohort.tw. | 85 |
|  | 95 | 69 or 70 or 71 or 72 or 73 or 74 or 75 or 76 or 77 or 78 or 79 or 80 or 81 or 82 or 83 or 84 or 85 or 86 or 87 or 88 or 89 or 90 or 91 or 92 or 93 or 94 | 3188283 |
|  | 96 | 41 and 63 and 68 and 95 | 544 |
|  | 97 | limit 96 to human | 386 |
|  | 98 | limit 97 to yr="2014 -Current" | 58 |

Database: MEDLINE In process

Name of Host: OVID

Number of results:  **18 (10 from 2014-2015)**

Date searched: 20 August 2015

*CANNOT LIMIT SEARCH TO HUMANS

Kingsland

|  | 1 | sport*.mp. | 6505 |
| --- | --- | --- | --- |
|  | 2 | cricket*.mp. | 294 |
|  | 3 | netball*.mp. | 17 |
|  | 4 | rugby.mp. | 243 |
|  | 5 | canoe*.mp. | 42 |
|  | 6 | softball*.mp. | 42 |
|  | 7 | triathl*.mp. | 122 |
|  | 8 | (waterpolo or water polo).mp. | 28 |
|  | 9 | (waterski* or water ski*).mp. | 23 |
|  | 10 | australian rules football*.mp. | 19 |
|  | 11 | surfing.mp. | 73 |
|  | 12 | handball*.mp. | 95 |
|  | 13 | yacht*.mp. | 12 |
|  | 14 | rowing.mp. | 102 |
|  | 15 | boating.mp. | 23 |
|  | 16 | sailing.mp. | 65 |
|  | 17 | lawn bowls.mp. | 1 |
|  | 18 | bowling.mp. | 41 |
|  | 19 | ((horse or harness or dog or motor or auto or car) adj rac*).mp. | 42 |
|  | 20 | ((motor or auto) adj sport*).mp. | 2 |
|  | 21 | (motorcycl* or motor cycl*).mp. | 310 |
|  | 22 | archery.mp. | 8 |
|  | 23 | equestrian.mp. | 30 |
|  | 24 | shooting.mp. | 355 |
|  | 25 | hunting.mp. | 490 |
|  | 26 | lacrosse.mp. | 36 |
|  | 27 | polo.mp. | 221 |
|  | 28 | table tennis.mp. | 25 |
|  | 29 | badminton.mp. | 28 |
|  | 30 | squash.mp. | 236 |
|  | 31 | cycling.mp. | 5637 |
|  | 32 | Fitness Centers/ | 0 |
|  | 33 | fitness centre*.mp. | 6 |
|  | 34 | gym*.mp. | 789 |
|  | 35 | (sport* and (game* or event* or club* or arena* or field* or ground*)).mp. | 1241 |
|  | 36 | athlet*.mp. | 5003 |
|  | 37 | player*.mp. | 5028 |
|  | 38 | spectator*.mp. | 268 |
|  | 39 | fan*.mp. | 2576 |
|  | 40 | (sport* and member*).mp. | 160 |
|  | 41 | 1 or 2 or 3 or 4 or 5 or 6 or 7 or 8 or 9 or 10 or 11 or 12 or 13 or 14 or 15 or 16 or 17 or 18 or 19 or 20 or 21 or 22 or 23 or 24 or 25 or 26 or 27 or 28 or 29 or 30 or 31 or 32 or 33 or 34 or 35 or 36 or 37 or 38 or 39 or 40 | 24087 |
|  | 42 | exp Health Promotion/ | 0 |
|  | 43 | exp Public Health/ | 0 |
|  | 44 | Harm Reduction/ | 0 |
|  | 45 | (harm* adj3 minimi*).mp. | 129 |
|  | 46 | Health Policy.mp. | 1821 |
|  | 47 | Public Policy.mp. | 665 |
|  | 48 | program*.mp. | 61986 |
|  | 49 | intervention*.mp. | 71217 |
|  | 50 | Preventive Medicine.mp. | 648 |
|  | 51 | Health Education/ | 0 |
|  | 52 | Consumer Health Information.mp. | 61 |
|  | 53 | Patient Education.mp. | 1280 |
|  | 54 | environment*.mp. | 85734 |
|  | 55 | (responsible and (alcohol* and beverage*) and servic*).mp. | 1 |
|  | 56 | server training.mp. | 0 |
|  | 57 | enforcement.mp. | 931 |
|  | 58 | community action*.mp. | 44 |
|  | 59 | community mobili*.mp. | 97 |
|  | 60 | (alcohol* and control*).mp. | 4489 |
|  | 61 | strateg*.mp. | 85846 |
|  | 62 | server intervention*.mp. | 3 |
|  | 63 | 42 or 43 or 44 or 45 or 46 or 47 or 48 or 49 or 50 or 51 or 52 or 53 or 54 or 55 or 56 or 57 or 58 or 59 or 60 or 61 or 62 | 273165 |
|  | 64 | exp Alcohol Drinking/ | 0 |
|  | 65 | alcohol*.mp. | 25204 |
|  | 66 | (alcohol* and (drunk* or incident* or safety or offen* or abus* or disorder* or harm* or violen* or injur* or intoxicat* or assault*)).mp. | 5854 |
|  | 67 | ((drunk* or drink*) and driv*).mp. | 313 |
|  | 68 | 64 or 65 or 66 or 67 | 25334 |
|  | 69 | randomized controlled trial.mp. | 6539 |
|  | 70 | controlled clinical trial.mp. | 1135 |
|  | 71 | randomized.ab. | 31343 |
|  | 72 | randomised.ab. | 6439 |
|  | 73 | clinical trial.mp. | 10751 |
|  | 74 | clinical trials.ab. | 16406 |
|  | 75 | trial.tw. | 38389 |
|  | 76 | double blind.tw. | 6619 |
|  | 77 | single blind.tw. | 761 |
|  | 78 | experiment*.tw. | 211374 |
|  | 79 | (pretest or Pre test).tw. | 1492 |
|  | 80 | (posttest or post test).tw. | 1740 |
|  | 81 | (pre post or prepost).tw. | 917 |
|  | 82 | before after.tw. | 377 |
|  | 83 | (quasi randomised or quasi randomized or quazi randomised or quazi randomized).tw. | 261 |
|  | 84 | stepped wedge.tw. | 60 |
|  | 85 | preference trial.tw. | 9 |
|  | 86 | natural experiment.tw. | 118 |
|  | 87 | ((quasi or quazi) adj experiment*).tw. | 1127 |
|  | 88 | (staggered enrolment trial* or staggered enrollment trial*).tw. | 2 |
|  | 89 | (non randomized or non randomised or nonrandomized or nonrandomised).tw. | 1623 |
|  | 90 | interrupted time series.tw. | 183 |
|  | 91 | (time series and trial).tw. | 73 |
|  | 92 | multiple baseline.tw. | 175 |
|  | 93 | regression discontinuity.tw. | 27 |
|  | 94 | comprehensive cohort.tw. | 6 |
|  | 95 | 69 or 70 or 71 or 72 or 73 or 74 or 75 or 76 or 77 or 78 or 79 or 80 or 81 or 82 or 83 or 84 or 85 or 86 or 87 or 88 or 89 or 90 or 91 or 92 or 93 or 94 | 279015 |
|  | 96 | 41 and 63 and 68 and 95 | 18 |
|  | 97 | limit 96 to yr="2014 -Current" | 10 |

Database: PsycINFO

Name of Host: OVID

Number of results:  **104 (16 for 2014-15)**

Date searched: 20^th^ August 2015

Rowland.

PsycEXTRA=6 and 3 after 2014

|  | 1 | sport*.mp. | 27560 |
| --- | --- | --- | --- |
|  | 2 | cricket*.mp. | 1025 |
|  | 3 | netball*.mp. | 85 |
|  | 4 | rugby.mp. | 470 |
|  | 5 | canoe*.mp. | 167 |
|  | 6 | softball*.mp. | 265 |
|  | 7 | triathl*.mp. | 156 |
|  | 8 | (waterpolo or water polo).mp. | 44 |
|  | 9 | (waterski* or water ski*).mp. | 21 |
|  | 10 | australian rules football*.mp. | 41 |
|  | 11 | surfing.mp. | 359 |
|  | 12 | handball*.mp. | 284 |
|  | 13 | yacht*.mp. | 33 |
|  | 14 | rowing.mp. | 163 |
|  | 15 | boating.mp. | 71 |
|  | 16 | sailing.mp. | 261 |
|  | 17 | lawn bowls.mp. | 4 |
|  | 18 | bowling.mp. | 362 |
|  | 19 | ((horse or harness or dog or motor or auto or car) adj rac*).mp. | 302 |
|  | 20 | ((motor or auto) adj sport*).mp. | 23 |
|  | 21 | (motorcycl* or motor cycl*).mp. | 742 |
|  | 22 | archery.mp. | 88 |
|  | 23 | equestrian.mp. | 90 |
|  | 24 | shooting.mp. | 1714 |
|  | 25 | hunting.mp. | 1817 |
|  | 26 | lacrosse.mp. | 85 |
|  | 27 | polo.mp. | 114 |
|  | 28 | table tennis.mp. | 168 |
|  | 29 | badminton.mp. | 135 |
|  | 30 | squash.mp. | 85 |
|  | 31 | cycling.mp. | 3594 |
|  | 32 | Fitness Centers/ | 0 |
|  | 33 | fitness centre*.mp. | 31 |
|  | 34 | gym*.mp. | 2037 |
|  | 35 | (sport* and (game* or event* or club* or arena* or field* or ground*)).mp. | 8253 |
|  | 36 | athlet*.mp. | 20680 |
|  | 37 | player*.mp. | 13910 |
|  | 38 | spectator*.mp. | 1808 |
|  | 39 | fan*.mp. | 21903 |
|  | 40 | (sport* and member*).mp. | 1486 |
|  | 41 | 1 or 2 or 3 or 4 or 5 or 6 or 7 or 8 or 9 or 10 or 11 or 12 or 13 or 14 or 15 or 16 or 17 or 18 or 19 or 20 or 21 or 22 or 23 or 24 or 25 or 26 or 27 or 28 or 29 or 30 or 31 or 32 or 33 or 34 or 35 or 36 or 37 or 38 or 39 or 40 | 77125 |
|  | 42 | exp Health Promotion/ | 17551 |
|  | 43 | exp Public Health/ | 16942 |
|  | 44 | Harm Reduction/ | 2211 |
|  | 45 | (harm* adj3 minimi*).mp. | 684 |
|  | 46 | Health Policy.mp. | 5162 |
|  | 47 | Public Policy.mp. | 8627 |
|  | 48 | program*.mp. | 335591 |
|  | 49 | intervention*.mp. | 273501 |
|  | 50 | Preventive Medicine.mp. | 2273 |
|  | 51 | Health Education/ | 10616 |
|  | 52 | Consumer Health Information.mp. | 58 |
|  | 53 | Patient Education.mp. | 2565 |
|  | 54 | environment*.mp. | 298258 |
|  | 55 | (responsible and (alcohol* and beverage*) and servic*).mp. | 53 |
|  | 56 | server training.mp. | 18 |
|  | 57 | enforcement.mp. | 9360 |
|  | 58 | community action*.mp. | 619 |
|  | 59 | community mobili*.mp. | 382 |
|  | 60 | (alcohol* and control*).mp. | 23480 |
|  | 61 | strateg*.mp. | 258038 |
|  | 62 | server intervention*.mp. | 27 |
|  | 63 | 42 or 43 or 44 or 45 or 46 or 47 or 48 or 49 or 50 or 51 or 52 or 53 or 54 or 55 or 56 or 57 or 58 or 59 or 60 or 61 or 62 | 995686 |
|  | 64 | exp Alcohol Drinking/ | 0 |
|  | 65 | alcohol*.mp. | 112224 |
|  | 66 | (alcohol* and (drunk* or incident* or safety or offen* or abus* or disorder* or harm* or violen* or injur* or intoxicat* or assault*)).mp. | 69254 |
|  | 67 | ((drunk* or drink*) and driv*).mp. | 3598 |
|  | 68 | 64 or 65 or 66 or 67 | 113080 |
|  | 69 | randomized controlled trial.mp. | 10269 |
|  | 70 | controlled clinical trial.mp. | 1090 |
|  | 71 | randomized.ab. | 44803 |
|  | 72 | randomised.ab. | 5531 |
|  | 73 | clinical trial.mp. | 10070 |
|  | 74 | randomly.ab. | 55552 |
|  | 75 | trial.tw. | 77473 |
|  | 76 | double blind.tw. | 19126 |
|  | 77 | single blind.tw. | 1430 |
|  | 78 | experiment*.tw. | 345876 |
|  | 79 | (pretest or Pre test).tw. | 13687 |
|  | 80 | (posttest or post test).tw. | 18283 |
|  | 81 | (pre post or prepost).tw. | 4376 |
|  | 82 | before after.tw. | 1277 |
|  | 83 | (quasi randomised or quasi randomized or quazi randomised or quazi randomized).tw. | 125 |
|  | 84 | stepped wedge.tw. | 17 |
|  | 85 | preference trial.tw. | 27 |
|  | 86 | natural experiment.tw. | 603 |
|  | 87 | ((quasi or quazi) adj experiment*).tw. | 7721 |
|  | 88 | (staggered enrolment trial* or staggered enrollment trial*).tw. | 0 |
|  | 89 | (non randomized or non randomised or nonrandomized or nonrandomised).tw. | 1425 |
|  | 90 | interrupted time series.tw. | 512 |
|  | 91 | (time series and trial).tw. | 117 |
|  | 92 | multiple baseline.tw. | 4104 |
|  | 93 | regression discontinuity.tw. | 201 |
|  | 94 | comprehensive cohort.tw. | 10 |
|  | 95 | 69 or 70 or 71 or 72 or 73 or 74 or 75 or 76 or 77 or 78 or 79 or 80 or 81 or 82 or 83 or 84 or 85 or 86 or 87 or 88 or 89 or 90 or 91 or 92 or 93 or 94 | 495703 |
|  | 96 | 41 and 63 and 68 and 95 | 104 |
|  | 97 | limit 96 to humans [Limit not valid in PsycINFO; records were retained] | 104 |
|  | 98 | limit 97 to yr="2014 -Current" | 16 |

Database: CINAHL

Name of Host: EBSCO

Number of results:  **51 (4 for 2014-15)**

Date searched: 20^th^ August 2015

Unsure: Tindall

|  | S98 | S41 AND S63 AND S68 AND S95  Limiters - Published Date: 20140501-20150831; Human | (4) | |
| --- | --- | --- | --- | --- |
|  | S97 | S41 AND S63 AND S68 AND S95  Limiters - Human | (51) |  |
|  | S96 | S41 AND S63 AND S68 AND S95 | [View Results](javascript:__doPostBack('ctl00$ctl00$FindField$FindField$historyControl$HistoryRepeater$ctl02$linkResults','')) (63) |  |
|  | S95 | S69 OR S70 OR S71 OR S72 OR S73 OR S74 OR S75 OR S76 OR S77 OR S78 OR S79 OR S80 OR S81 OR S82 OR S83 OR S84 OR S85 OR S86 OR S87 OR S88 OR S89 OR S90 OR S91 OR S92 OR S93 OR S94 | [View Results](javascript:__doPostBack('ctl00$ctl00$FindField$FindField$historyControl$HistoryRepeater$ctl03$linkResults','')) (372,240) |  |
|  | S94 | comprehensive cohort | [View Results](javascript:__doPostBack('ctl00$ctl00$FindField$FindField$historyControl$HistoryRepeater$ctl04$linkResults','')) (72) |  |
|  | S93 | regression discontinuity | [View Results](javascript:__doPostBack('ctl00$ctl00$FindField$FindField$historyControl$HistoryRepeater$ctl05$linkResults','')) (41) |  |
|  | S92 | multiple baseline | [View Results](javascript:__doPostBack('ctl00$ctl00$FindField$FindField$historyControl$HistoryRepeater$ctl06$linkResults','')) (913) |  |
|  | S91 | (time series and trial) | [View Results](javascript:__doPostBack('ctl00$ctl00$FindField$FindField$historyControl$HistoryRepeater$ctl07$linkResults','')) (194) |  |
|  | S90 | interrupted time series | [View Results](javascript:__doPostBack('ctl00$ctl00$FindField$FindField$historyControl$HistoryRepeater$ctl08$linkResults','')) (603) |  |
|  | S89 | (non randomized or non randomised or nonrandomized or nonrandomised) | [View Results](javascript:__doPostBack('ctl00$ctl00$FindField$FindField$historyControl$HistoryRepeater$ctl09$linkResults','')) (4,264) |  |
|  | S88 | (staggered enrolment trial* or staggered enrollment trial*) | [View Results](javascript:__doPostBack('ctl00$ctl00$FindField$FindField$historyControl$HistoryRepeater$ctl10$linkResults','')) (0) |  |
|  | S87 | ((quasi or quazi) n1 experiment*) | [View Results](javascript:__doPostBack('ctl00$ctl00$FindField$FindField$historyControl$HistoryRepeater$ctl11$linkResults','')) (8,813) |  |
|  | S86 | natural experiment | [View Results](javascript:__doPostBack('ctl00$ctl00$FindField$FindField$historyControl$HistoryRepeater$ctl12$linkResults','')) (302) |  |
|  | S85 | preference trial | [View Results](javascript:__doPostBack('ctl00$ctl00$FindField$FindField$historyControl$HistoryRepeater$ctl13$linkResults','')) (71) |  |
|  | S84 | stepped wedge | [View Results](javascript:__doPostBack('ctl00$ctl00$FindField$FindField$historyControl$HistoryRepeater$ctl14$linkResults','')) (80) |  |
|  | S83 | (quasi randomised or quasi randomized or quazi randomised or quazi randomized) | [View Results](javascript:__doPostBack('ctl00$ctl00$FindField$FindField$historyControl$HistoryRepeater$ctl15$linkResults','')) (1,792) |  |
|  | S82 | before after | [View Results](javascript:__doPostBack('ctl00$ctl00$FindField$FindField$historyControl$HistoryRepeater$ctl16$linkResults','')) (35,117) |  |
|  | S81 | (pre post or prepost) | [View Results](javascript:__doPostBack('ctl00$ctl00$FindField$FindField$historyControl$HistoryRepeater$ctl17$linkResults','')) (12,653) |  |
|  | S80 | (posttest or post test) | [View Results](javascript:__doPostBack('ctl00$ctl00$FindField$FindField$historyControl$HistoryRepeater$ctl18$linkResults','')) (33,966) |  |
|  | S79 | (pretest or pre test) | [View Results](javascript:__doPostBack('ctl00$ctl00$FindField$FindField$historyControl$HistoryRepeater$ctl19$linkResults','')) (32,853) |  |
|  | S78 | experiment* | [View Results](javascript:__doPostBack('ctl00$ctl00$FindField$FindField$historyControl$HistoryRepeater$ctl20$linkResults','')) (73,339) |  |
|  | S77 | single blind | [View Results](javascript:__doPostBack('ctl00$ctl00$FindField$FindField$historyControl$HistoryRepeater$ctl21$linkResults','')) (10,548) |  |
|  | S76 | double blind | [View Results](javascript:__doPostBack('ctl00$ctl00$FindField$FindField$historyControl$HistoryRepeater$ctl22$linkResults','')) (37,360) |  |
|  | S75 | trial | [View Results](javascript:__doPostBack('ctl00$ctl00$FindField$FindField$historyControl$HistoryRepeater$ctl23$linkResults','')) (100,521) |  |
|  | S74 | AB clinical trials | [View Results](javascript:__doPostBack('ctl00$ctl00$FindField$FindField$historyControl$HistoryRepeater$ctl24$linkResults','')) (22,987) |  |
|  | S73 | controlled clinical trial | [View Results](javascript:__doPostBack('ctl00$ctl00$FindField$FindField$historyControl$HistoryRepeater$ctl25$linkResults','')) (3,459) |  |
|  | S72 | AB randomised | [View Results](javascript:__doPostBack('ctl00$ctl00$FindField$FindField$historyControl$HistoryRepeater$ctl26$linkResults','')) (15,686) |  |
|  | S71 | AB randomized | [View Results](javascript:__doPostBack('ctl00$ctl00$FindField$FindField$historyControl$HistoryRepeater$ctl27$linkResults','')) (59,716) |  |
|  | S70 | (MH "Clinical Trials") | [View Results](javascript:__doPostBack('ctl00$ctl00$FindField$FindField$historyControl$HistoryRepeater$ctl28$linkResults','')) (122,631) |  |
|  | S69 | (MH " Randomized Controlled Trials ") | [View Results](javascript:__doPostBack('ctl00$ctl00$FindField$FindField$historyControl$HistoryRepeater$ctl29$linkResults','')) (39,195) |  |
|  | S68 | S64 OR S65 OR S66 OR S67 | [View Results](javascript:__doPostBack('ctl00$ctl00$FindField$FindField$historyControl$HistoryRepeater$ctl30$linkResults','')) (60,347) |  |
|  | S67 | ((drunk* or drink*) and driv*) | [View Results](javascript:__doPostBack('ctl00$ctl00$FindField$FindField$historyControl$HistoryRepeater$ctl31$linkResults','')) (1,369) |  |
|  | S66 | (alcohol* and (drunk* or incident* or safety or offen* or abus* or disorder* or harm* or violen* or injur* or intoxicat* or assault*)) | [View Results](javascript:__doPostBack('ctl00$ctl00$FindField$FindField$historyControl$HistoryRepeater$ctl32$linkResults','')) (30,473) |  |
|  | S65 | alcohol* | [View Results](javascript:__doPostBack('ctl00$ctl00$FindField$FindField$historyControl$HistoryRepeater$ctl33$linkResults','')) (60,183) |  |
|  | S64 | exp Alcohol Drinking/ | [View Results](javascript:__doPostBack('ctl00$ctl00$FindField$FindField$historyControl$HistoryRepeater$ctl34$linkResults','')) (2) |  |
|  | S63 | S42 OR S43 OR S44 OR S45 OR S46 OR S47 OR S48 OR S49 OR S50 OR S51 OR S52 OR S53 OR S54 OR S55 OR S56 OR S57 OR S58 OR S59 OR S60 OR S61 OR S62 | [View Results](javascript:__doPostBack('ctl00$ctl00$FindField$FindField$historyControl$HistoryRepeater$ctl35$linkResults','')) (1,347,002) |  |
|  | S62 | (MH "Consumer Health Information") | [View Results](javascript:__doPostBack('ctl00$ctl00$FindField$FindField$historyControl$HistoryRepeater$ctl36$linkResults','')) (9,657) |  |
|  | S61 | (MH "Patient Education") | [View Results](javascript:__doPostBack('ctl00$ctl00$FindField$FindField$historyControl$HistoryRepeater$ctl37$linkResults','')) (51,296) |  |
|  | S60 | strateg* | [View Results](javascript:__doPostBack('ctl00$ctl00$FindField$FindField$historyControl$HistoryRepeater$ctl38$linkResults','')) (133,868) |  |
|  | S59 | (alcohol* and control*) | [View Results](javascript:__doPostBack('ctl00$ctl00$FindField$FindField$historyControl$HistoryRepeater$ctl39$linkResults','')) (17,363) |  |
|  | S58 | community mobili* | [View Results](javascript:__doPostBack('ctl00$ctl00$FindField$FindField$historyControl$HistoryRepeater$ctl40$linkResults','')) (788) |  |
|  | S57 | community action* | [View Results](javascript:__doPostBack('ctl00$ctl00$FindField$FindField$historyControl$HistoryRepeater$ctl41$linkResults','')) (914) |  |
|  | S56 | enforcement | [View Results](javascript:__doPostBack('ctl00$ctl00$FindField$FindField$historyControl$HistoryRepeater$ctl42$linkResults','')) (2,823) |  |
|  | S55 | server intervention* | [View Results](javascript:__doPostBack('ctl00$ctl00$FindField$FindField$historyControl$HistoryRepeater$ctl43$linkResults','')) (12) |  |
|  | S54 | server training | [View Results](javascript:__doPostBack('ctl00$ctl00$FindField$FindField$historyControl$HistoryRepeater$ctl44$linkResults','')) (11) |  |
|  | S53 | (responsible and (alcohol* or beverage*) and servic*) | [View Results](javascript:__doPostBack('ctl00$ctl00$FindField$FindField$historyControl$HistoryRepeater$ctl45$linkResults','')) (95) |  |
|  | S52 | environment* | [View Results](javascript:__doPostBack('ctl00$ctl00$FindField$FindField$historyControl$HistoryRepeater$ctl46$linkResults','')) (139,485) |  |
|  | S51 | (MH "Health Education") | [View Results](javascript:__doPostBack('ctl00$ctl00$FindField$FindField$historyControl$HistoryRepeater$ctl47$linkResults','')) (19,137) |  |
|  | S50 | (MH "Health Policy") | [View Results](javascript:__doPostBack('ctl00$ctl00$FindField$FindField$historyControl$HistoryRepeater$ctl48$linkResults','')) (36,198) |  |
|  | S49 | (MH "Preventive Health Care") OR "Preventive Medicine" | [View Results](javascript:__doPostBack('ctl00$ctl00$FindField$FindField$historyControl$HistoryRepeater$ctl49$linkResults','')) (15,264) |  |
|  | S48 | intervention* | (216,629) |  |
|  | S47 | program* | [View Results](javascript:__doPostBack('ctl00$ctl00$FindField$FindField$historyControl$HistoryRepeater$ctl51$linkResults','')) (301,332) |  |
|  | S46 | (MH "Public Policy") | [View Results](javascript:__doPostBack('ctl00$ctl00$FindField$FindField$historyControl$HistoryRepeater$ctl52$linkResults','')) (15,016) |  |
|  | S45 | (harm* n3 minimi*) | [View Results](javascript:__doPostBack('ctl00$ctl00$FindField$FindField$historyControl$HistoryRepeater$ctl53$linkResults','')) (389) |  |
|  | S44 | (MH "Harm Reduction") | [View Results](javascript:__doPostBack('ctl00$ctl00$FindField$FindField$historyControl$HistoryRepeater$ctl54$linkResults','')) (2,039) |  |
|  | S43 | (MH "Public Health+") | [View Results](javascript:__doPostBack('ctl00$ctl00$FindField$FindField$historyControl$HistoryRepeater$ctl55$linkResults','')) (741,317) |  |
|  | S42 | (MH "Health Promotion+") | [View Results](javascript:__doPostBack('ctl00$ctl00$FindField$FindField$historyControl$HistoryRepeater$ctl56$linkResults','')) (42,838) |  |
|  | S41 | S1 OR S2 OR S3 OR S4 OR S5 OR S6 OR S7 OR S8 OR S9 OR S10 OR S11 OR S12 OR S13 OR S14 OR S15 OR S16 OR S17 OR S18 OR S19 OR S20 OR S21 OR S22 OR S23 OR S24 OR S25 OR S26 OR S27 OR S28 OR S29 OR S30 OR S31 OR S32 OR S33 OR S34 OR S35 OR S36 OR S37 OR S38 OR S39 OR S40 | [View Results](javascript:__doPostBack('ctl00$ctl00$FindField$FindField$historyControl$HistoryRepeater$ctl57$linkResults','')) (78,181) |  |
|  | S40 | hunting | [View Results](javascript:__doPostBack('ctl00$ctl00$FindField$FindField$historyControl$HistoryRepeater$ctl58$linkResults','')) (398) |  |
|  | S39 | surfing | [View Results](javascript:__doPostBack('ctl00$ctl00$FindField$FindField$historyControl$HistoryRepeater$ctl59$linkResults','')) (387) |  |
|  | S38 | badminton | [View Results](javascript:__doPostBack('ctl00$ctl00$FindField$FindField$historyControl$HistoryRepeater$ctl60$linkResults','')) (101) |  |
|  | S37 | polo | [View Results](javascript:__doPostBack('ctl00$ctl00$FindField$FindField$historyControl$HistoryRepeater$ctl61$linkResults','')) (183) |  |
|  | S36 | (sport* and member*) | [View Results](javascript:__doPostBack('ctl00$ctl00$FindField$FindField$historyControl$HistoryRepeater$ctl62$linkResults','')) (660) |  |
|  | S35 | fan* | [View Results](javascript:__doPostBack('ctl00$ctl00$FindField$FindField$historyControl$HistoryRepeater$ctl63$linkResults','')) (2,791) |  |
|  | S34 | spectator* | [View Results](javascript:__doPostBack('ctl00$ctl00$FindField$FindField$historyControl$HistoryRepeater$ctl64$linkResults','')) (167) |  |
|  | S33 | player* | [View Results](javascript:__doPostBack('ctl00$ctl00$FindField$FindField$historyControl$HistoryRepeater$ctl65$linkResults','')) (9,332) |  |
|  | S32 | (MH "Athletes") OR "athlet*" | [View Results](javascript:__doPostBack('ctl00$ctl00$FindField$FindField$historyControl$HistoryRepeater$ctl66$linkResults','')) (42,079) |  |
|  | S31 | (sport* and (game* or event* or club* or arena* or field* or ground*)) | [View Results](javascript:__doPostBack('ctl00$ctl00$FindField$FindField$historyControl$HistoryRepeater$ctl67$linkResults','')) (8,352) |  |
|  | S30 | gym* | [View Results](javascript:__doPostBack('ctl00$ctl00$FindField$FindField$historyControl$HistoryRepeater$ctl68$linkResults','')) (1,667) |  |
|  | S29 | fitness centre* | [View Results](javascript:__doPostBack('ctl00$ctl00$FindField$FindField$historyControl$HistoryRepeater$ctl69$linkResults','')) (45) |  |
|  | S28 | (MH "Fitness Centers") | [View Results](javascript:__doPostBack('ctl00$ctl00$FindField$FindField$historyControl$HistoryRepeater$ctl70$linkResults','')) (1,109) |  |
|  | S27 | cycling | [View Results](javascript:__doPostBack('ctl00$ctl00$FindField$FindField$historyControl$HistoryRepeater$ctl71$linkResults','')) (7,885) |  |
|  | S26 | squash | [View Results](javascript:__doPostBack('ctl00$ctl00$FindField$FindField$historyControl$HistoryRepeater$ctl72$linkResults','')) (259) |  |
|  | S25 | table tennis | [View Results](javascript:__doPostBack('ctl00$ctl00$FindField$FindField$historyControl$HistoryRepeater$ctl73$linkResults','')) (56) |  |
|  | S24 | lacrosse | [View Results](javascript:__doPostBack('ctl00$ctl00$FindField$FindField$historyControl$HistoryRepeater$ctl74$linkResults','')) (136) |  |
|  | S23 | shooting | [View Results](javascript:__doPostBack('ctl00$ctl00$FindField$FindField$historyControl$HistoryRepeater$ctl75$linkResults','')) (668) |  |
|  | S22 | equestrian | [View Results](javascript:__doPostBack('ctl00$ctl00$FindField$FindField$historyControl$HistoryRepeater$ctl76$linkResults','')) (45) |  |
|  | S21 | archery | [View Results](javascript:__doPostBack('ctl00$ctl00$FindField$FindField$historyControl$HistoryRepeater$ctl77$linkResults','')) (43) |  |
|  | S20 | (motorcycl* or motor cycl*) | [View Results](javascript:__doPostBack('ctl00$ctl00$FindField$FindField$historyControl$HistoryRepeater$ctl78$linkResults','')) (827) |  |
|  | S19 | ((motor or auto) n1 sport*) | [View Results](javascript:__doPostBack('ctl00$ctl00$FindField$FindField$historyControl$HistoryRepeater$ctl79$linkResults','')) (243) |  |
|  | S18 | ((horse or harness or dog or motor or auto or car) n1 rac*) | [View Results](javascript:__doPostBack('ctl00$ctl00$FindField$FindField$historyControl$HistoryRepeater$ctl80$linkResults','')) (100) |  |
|  | S17 | bowling | [View Results](javascript:__doPostBack('ctl00$ctl00$FindField$FindField$historyControl$HistoryRepeater$ctl81$linkResults','')) (251) |  |
|  | S16 | lawn bowls | [View Results](javascript:__doPostBack('ctl00$ctl00$FindField$FindField$historyControl$HistoryRepeater$ctl82$linkResults','')) (3) |  |
|  | S15 | sailing | [View Results](javascript:__doPostBack('ctl00$ctl00$FindField$FindField$historyControl$HistoryRepeater$ctl83$linkResults','')) (253) |  |
|  | S14 | boating | [View Results](javascript:__doPostBack('ctl00$ctl00$FindField$FindField$historyControl$HistoryRepeater$ctl84$linkResults','')) (57) |  |
|  | S13 | rowing | [View Results](javascript:__doPostBack('ctl00$ctl00$FindField$FindField$historyControl$HistoryRepeater$ctl85$linkResults','')) (774) |  |
|  | S12 | yacht* | [View Results](javascript:__doPostBack('ctl00$ctl00$FindField$FindField$historyControl$HistoryRepeater$ctl86$linkResults','')) (35) |  |
|  | S11 | handball* | [View Results](javascript:__doPostBack('ctl00$ctl00$FindField$FindField$historyControl$HistoryRepeater$ctl87$linkResults','')) (314) |  |
|  | S10 | australian rules football* | [View Results](javascript:__doPostBack('ctl00$ctl00$FindField$FindField$historyControl$HistoryRepeater$ctl88$linkResults','')) (82) |  |
|  | S9 | (waterski* or water ski*) | [View Results](javascript:__doPostBack('ctl00$ctl00$FindField$FindField$historyControl$HistoryRepeater$ctl89$linkResults','')) (286) |  |
|  | S8 | (waterpolo or water polo) | [View Results](javascript:__doPostBack('ctl00$ctl00$FindField$FindField$historyControl$HistoryRepeater$ctl90$linkResults','')) (115) |  |
|  | S7 | triathl* | [View Results](javascript:__doPostBack('ctl00$ctl00$FindField$FindField$historyControl$HistoryRepeater$ctl91$linkResults','')) (735) |  |
|  | S6 | softball* | [View Results](javascript:__doPostBack('ctl00$ctl00$FindField$FindField$historyControl$HistoryRepeater$ctl92$linkResults','')) (235) |  |
|  | S5 | canoe* | [View Results](javascript:__doPostBack('ctl00$ctl00$FindField$FindField$historyControl$HistoryRepeater$ctl93$linkResults','')) (92) |  |
|  | S4 | rugby | [View Results](javascript:__doPostBack('ctl00$ctl00$FindField$FindField$historyControl$HistoryRepeater$ctl94$linkResults','')) (1,378) |  |
|  | S3 | netball* | [View Results](javascript:__doPostBack('ctl00$ctl00$FindField$FindField$historyControl$HistoryRepeater$ctl95$linkResults','')) (77) |  |
|  | S2 | cricket | [View Results](javascript:__doPostBack('ctl00$ctl00$FindField$FindField$historyControl$HistoryRepeater$ctl96$linkResults','')) (287) |  |
|  | S1 | sport* |  |  |

Database: SPORTDISCUS

Name of Host: EBSCO

Number of results:  **87 (12 from 2014-15)**

Date searched: 20^th^ August 2015

CANNOT LIMIT TO HUMANS

Nicholsons

Tindall

|  | S98 | S41 AND S63 AND S68 AND S95  Limiters - Published Date: 20140501-20150831 | [View Results](javascript:__doPostBack('ctl00$ctl00$MainContentArea$MainContentArea$historyControl$HistoryRepeater$ctl98$linkResults','')) (12) |
| --- | --- | --- | --- |
|  | S97 | S41 AND S63 AND S68 AND S95  Limiters-Human | [View Results](javascript:__doPostBack('ctl00$ctl00$MainContentArea$MainContentArea$historyControl$HistoryRepeater$ctl99$linkResults','')) (87) |
|  | S96 | S41 AND S63 AND S68 AND S95 | [View Results](javascript:__doPostBack('ctl00$ctl00$MainContentArea$MainContentArea$historyControl$HistoryRepeater$ctl100$linkResults','')) (87) |
|  | S95 | S69 OR S70 OR S71 OR S72 OR S73 OR S74 OR S75 OR S76 OR S77 OR S78 OR S79 OR S80 OR S81 OR S82 OR S83 OR S84 OR S85 OR S86 OR S87 OR S88 OR S89 OR S90 OR S91 OR S92 OR S93 OR S94 | [View Results](javascript:__doPostBack('ctl00$ctl00$MainContentArea$MainContentArea$historyControl$HistoryRepeater$ctl101$linkResults','')) (103,056) |
|  | S94 | comprehensive cohort | [View Results](javascript:__doPostBack('ctl00$ctl00$MainContentArea$MainContentArea$historyControl$HistoryRepeater$ctl102$linkResults','')) (10) |
|  | S93 | regression discontinuity | [View Results](javascript:__doPostBack('ctl00$ctl00$MainContentArea$MainContentArea$historyControl$HistoryRepeater$ctl103$linkResults','')) (6) |
|  | S92 | multiple baseline | [View Results](javascript:__doPostBack('ctl00$ctl00$MainContentArea$MainContentArea$historyControl$HistoryRepeater$ctl104$linkResults','')) (249) |
|  | S91 | (time series and trial) | [View Results](javascript:__doPostBack('ctl00$ctl00$MainContentArea$MainContentArea$historyControl$HistoryRepeater$ctl105$linkResults','')) (168) |
|  | S90 | interrupted time series | [View Results](javascript:__doPostBack('ctl00$ctl00$MainContentArea$MainContentArea$historyControl$HistoryRepeater$ctl106$linkResults','')) (68) |
|  | S89 | (non randomized or non randomised or nonrandomized or nonrandomised) | [View Results](javascript:__doPostBack('ctl00$ctl00$MainContentArea$MainContentArea$historyControl$HistoryRepeater$ctl107$linkResults','')) (600) |
|  | S88 | (staggered enrolment trial* or staggered enrollment trial*) | [View Results](javascript:__doPostBack('ctl00$ctl00$MainContentArea$MainContentArea$historyControl$HistoryRepeater$ctl108$linkResults','')) (0) |
|  | S87 | ((quasi or quazi) n1 experiment*) | [View Results](javascript:__doPostBack('ctl00$ctl00$MainContentArea$MainContentArea$historyControl$HistoryRepeater$ctl109$linkResults','')) (712) |
|  | S86 | natural experiment | [View Results](javascript:__doPostBack('ctl00$ctl00$MainContentArea$MainContentArea$historyControl$HistoryRepeater$ctl110$linkResults','')) (113) |
|  | S85 | preference trial | [View Results](javascript:__doPostBack('ctl00$ctl00$MainContentArea$MainContentArea$historyControl$HistoryRepeater$ctl111$linkResults','')) (31) |
|  | S84 | stepped wedge | [View Results](javascript:__doPostBack('ctl00$ctl00$MainContentArea$MainContentArea$historyControl$HistoryRepeater$ctl112$linkResults','')) (1) |
|  | S83 | (quasi randomised or quasi randomized or quazi randomised or quazi randomized) | [View Results](javascript:__doPostBack('ctl00$ctl00$MainContentArea$MainContentArea$historyControl$HistoryRepeater$ctl113$linkResults','')) (105) |
|  | S82 | before after | [View Results](javascript:__doPostBack('ctl00$ctl00$MainContentArea$MainContentArea$historyControl$HistoryRepeater$ctl114$linkResults','')) (14,863) |
|  | S81 | (pre post or prepost) | [View Results](javascript:__doPostBack('ctl00$ctl00$MainContentArea$MainContentArea$historyControl$HistoryRepeater$ctl115$linkResults','')) (7,405) |
|  | S80 | (posttest or post test) | [View Results](javascript:__doPostBack('ctl00$ctl00$MainContentArea$MainContentArea$historyControl$HistoryRepeater$ctl116$linkResults','')) (5,662) |
|  | S79 | (pretest or pre test) | [View Results](javascript:__doPostBack('ctl00$ctl00$MainContentArea$MainContentArea$historyControl$HistoryRepeater$ctl117$linkResults','')) (4,903) |
|  | S78 | experiment* | [View Results](javascript:__doPostBack('ctl00$ctl00$MainContentArea$MainContentArea$historyControl$HistoryRepeater$ctl118$linkResults','')) (45,735) |
|  | S77 | single blind | [View Results](javascript:__doPostBack('ctl00$ctl00$MainContentArea$MainContentArea$historyControl$HistoryRepeater$ctl119$linkResults','')) (660) |
|  | S76 | double blind | [View Results](javascript:__doPostBack('ctl00$ctl00$MainContentArea$MainContentArea$historyControl$HistoryRepeater$ctl120$linkResults','')) (4,497) |
|  | S75 | trial | [View Results](javascript:__doPostBack('ctl00$ctl00$MainContentArea$MainContentArea$historyControl$HistoryRepeater$ctl121$linkResults','')) (41,003) |
|  | S74 | clinical trials | [View Results](javascript:__doPostBack('ctl00$ctl00$MainContentArea$MainContentArea$historyControl$HistoryRepeater$ctl122$linkResults','')) (11,094) |
|  | S73 | controlled clinical trial | [View Results](javascript:__doPostBack('ctl00$ctl00$MainContentArea$MainContentArea$historyControl$HistoryRepeater$ctl123$linkResults','')) (922) |
|  | S72 | randomised | [View Results](javascript:__doPostBack('ctl00$ctl00$MainContentArea$MainContentArea$historyControl$HistoryRepeater$ctl124$linkResults','')) (3,099) |
|  | S71 | randomized | [View Results](javascript:__doPostBack('ctl00$ctl00$MainContentArea$MainContentArea$historyControl$HistoryRepeater$ctl125$linkResults','')) (16,381) |
|  | S70 | Clinical Trials | [View Results](javascript:__doPostBack('ctl00$ctl00$MainContentArea$MainContentArea$historyControl$HistoryRepeater$ctl126$linkResults','')) (11,094) |
|  | S69 | Randomized Controlled Trials | [View Results](javascript:__doPostBack('ctl00$ctl00$MainContentArea$MainContentArea$historyControl$HistoryRepeater$ctl127$linkResults','')) (8,873) |
|  | S68 | S64 OR S65 OR S66 OR S67 | [View Results](javascript:__doPostBack('ctl00$ctl00$MainContentArea$MainContentArea$historyControl$HistoryRepeater$ctl128$linkResults','')) (18,239) |
|  | S67 | ((drunk* or drink*) and driv*) | [View Results](javascript:__doPostBack('ctl00$ctl00$MainContentArea$MainContentArea$historyControl$HistoryRepeater$ctl129$linkResults','')) (939) |
|  | S66 | (alcohol* and (drunk* or incident* or safety or offen* or abus* or disorder* or harm* or violen* or injur* or intoxicat* or assault*)) | [View Results](javascript:__doPostBack('ctl00$ctl00$MainContentArea$MainContentArea$historyControl$HistoryRepeater$ctl130$linkResults','')) (9,755) |
|  | S65 | alcohol* | [View Results](javascript:__doPostBack('ctl00$ctl00$MainContentArea$MainContentArea$historyControl$HistoryRepeater$ctl131$linkResults','')) (17,863) |
|  | S64 | exp Alcohol Drinking/ | [View Results](javascript:__doPostBack('ctl00$ctl00$MainContentArea$MainContentArea$historyControl$HistoryRepeater$ctl132$linkResults','')) (1) |
|  | S63 | S42 OR S43 OR S44 OR S45 OR S46 OR S47 OR S48 OR S49 OR S50 OR S51 OR S52 OR S53 OR S54 OR S55 OR S56 OR S57 OR S58 OR S59 OR S60 OR S61 OR S62 | [View Results](javascript:__doPostBack('ctl00$ctl00$MainContentArea$MainContentArea$historyControl$HistoryRepeater$ctl133$linkResults','')) (305,958) |
|  | S62 | Consumer Health Information | [View Results](javascript:__doPostBack('ctl00$ctl00$MainContentArea$MainContentArea$historyControl$HistoryRepeater$ctl134$linkResults','')) (64) |
|  | S61 | Patient Education | [View Results](javascript:__doPostBack('ctl00$ctl00$MainContentArea$MainContentArea$historyControl$HistoryRepeater$ctl135$linkResults','')) (1,384) |
|  | S60 | strateg* | [View Results](javascript:__doPostBack('ctl00$ctl00$MainContentArea$MainContentArea$historyControl$HistoryRepeater$ctl136$linkResults','')) (54,709) |
|  | S59 | (alcohol* and control*) | [View Results](javascript:__doPostBack('ctl00$ctl00$MainContentArea$MainContentArea$historyControl$HistoryRepeater$ctl137$linkResults','')) (3,242) |
|  | S58 | community mobili* | [View Results](javascript:__doPostBack('ctl00$ctl00$MainContentArea$MainContentArea$historyControl$HistoryRepeater$ctl138$linkResults','')) (260) |
|  | S57 | community action* | [View Results](javascript:__doPostBack('ctl00$ctl00$MainContentArea$MainContentArea$historyControl$HistoryRepeater$ctl139$linkResults','')) (300) |
|  | S56 | enforcement | [View Results](javascript:__doPostBack('ctl00$ctl00$MainContentArea$MainContentArea$historyControl$HistoryRepeater$ctl140$linkResults','')) (2,405) |
|  | S55 | server intervention* | [View Results](javascript:__doPostBack('ctl00$ctl00$MainContentArea$MainContentArea$historyControl$HistoryRepeater$ctl141$linkResults','')) (9) |
|  | S54 | server training | [View Results](javascript:__doPostBack('ctl00$ctl00$MainContentArea$MainContentArea$historyControl$HistoryRepeater$ctl142$linkResults','')) (15) |
|  | S53 | (responsible and (alcohol* or beverage*) and servic*) | [View Results](javascript:__doPostBack('ctl00$ctl00$MainContentArea$MainContentArea$historyControl$HistoryRepeater$ctl143$linkResults','')) (48) |
|  | S52 | environment* | [View Results](javascript:__doPostBack('ctl00$ctl00$MainContentArea$MainContentArea$historyControl$HistoryRepeater$ctl144$linkResults','')) (43,806) |
|  | S51 | Health Education | [View Results](javascript:__doPostBack('ctl00$ctl00$MainContentArea$MainContentArea$historyControl$HistoryRepeater$ctl145$linkResults','')) (24,982) |
|  | S50 | Health Policy | [View Results](javascript:__doPostBack('ctl00$ctl00$MainContentArea$MainContentArea$historyControl$HistoryRepeater$ctl146$linkResults','')) (4,238) |
|  | S49 | "Preventive Medicine" | [View Results](javascript:__doPostBack('ctl00$ctl00$MainContentArea$MainContentArea$historyControl$HistoryRepeater$ctl147$linkResults','')) (5,267) |
|  | S48 | intervention* | [View Results](javascript:__doPostBack('ctl00$ctl00$MainContentArea$MainContentArea$historyControl$HistoryRepeater$ctl148$linkResults','')) (37,976) |
|  | S47 | program* | [View Results](javascript:__doPostBack('ctl00$ctl00$MainContentArea$MainContentArea$historyControl$HistoryRepeater$ctl149$linkResults','')) (156,110) |
|  | S46 | Public Policy | [View Results](javascript:__doPostBack('ctl00$ctl00$MainContentArea$MainContentArea$historyControl$HistoryRepeater$ctl150$linkResults','')) (3,304) |
|  | S45 | (harm* n3 minimi*) | [View Results](javascript:__doPostBack('ctl00$ctl00$MainContentArea$MainContentArea$historyControl$HistoryRepeater$ctl151$linkResults','')) (170) |
|  | S44 | Harm Reduction | [View Results](javascript:__doPostBack('ctl00$ctl00$MainContentArea$MainContentArea$historyControl$HistoryRepeater$ctl152$linkResults','')) (731) |
|  | S43 | Public Health | [View Results](javascript:__doPostBack('ctl00$ctl00$MainContentArea$MainContentArea$historyControl$HistoryRepeater$ctl153$linkResults','')) (35,984) |
|  | S42 | Health Promotion | [View Results](javascript:__doPostBack('ctl00$ctl00$MainContentArea$MainContentArea$historyControl$HistoryRepeater$ctl154$linkResults','')) (13,908) |
|  | S41 | S1 OR S2 OR S3 OR S4 OR S5 OR S6 OR S7 OR S8 OR S9 OR S10 OR S11 OR S12 OR S13 OR S14 OR S15 OR S16 OR S17 OR S18 OR S19 OR S20 OR S21 OR S22 OR S23 OR S24 OR S25 OR S26 OR S27 OR S28 OR S29 OR S30 OR S31 OR S32 OR S33 OR S34 OR S35 OR S36 OR S37 OR S38 OR S39 OR S40 | [View Results](javascript:__doPostBack('ctl00$ctl00$MainContentArea$MainContentArea$historyControl$HistoryRepeater$ctl155$linkResults','')) (1,199,443) |
|  | S40 | hunting | [View Results](javascript:__doPostBack('ctl00$ctl00$MainContentArea$MainContentArea$historyControl$HistoryRepeater$ctl156$linkResults','')) (8,886) |
|  | S39 | surfing | [View Results](javascript:__doPostBack('ctl00$ctl00$MainContentArea$MainContentArea$historyControl$HistoryRepeater$ctl157$linkResults','')) (5,911) |
|  | S38 | badminton | [View Results](javascript:__doPostBack('ctl00$ctl00$MainContentArea$MainContentArea$historyControl$HistoryRepeater$ctl158$linkResults','')) (2,894) |
|  | S37 | polo | [View Results](javascript:__doPostBack('ctl00$ctl00$MainContentArea$MainContentArea$historyControl$HistoryRepeater$ctl159$linkResults','')) (3,639) |
|  | S36 | (sport* and member*) | [View Results](javascript:__doPostBack('ctl00$ctl00$MainContentArea$MainContentArea$historyControl$HistoryRepeater$ctl160$linkResults','')) (17,154) |
|  | S35 | fan* | [View Results](javascript:__doPostBack('ctl00$ctl00$MainContentArea$MainContentArea$historyControl$HistoryRepeater$ctl161$linkResults','')) (24,211) |
|  | S34 | spectator* | [View Results](javascript:__doPostBack('ctl00$ctl00$MainContentArea$MainContentArea$historyControl$HistoryRepeater$ctl162$linkResults','')) (9,020) |
|  | S33 | player* | [View Results](javascript:__doPostBack('ctl00$ctl00$MainContentArea$MainContentArea$historyControl$HistoryRepeater$ctl163$linkResults','')) (196,284) |
|  | S32 | (MH "Athletes") OR "athlet*" | [View Results](javascript:__doPostBack('ctl00$ctl00$MainContentArea$MainContentArea$historyControl$HistoryRepeater$ctl164$linkResults','')) (308,983) |
|  | S31 | (sport* and (game* or event* or club* or arena* or field* or ground*)) | [View Results](javascript:__doPostBack('ctl00$ctl00$MainContentArea$MainContentArea$historyControl$HistoryRepeater$ctl165$linkResults','')) (211,915) |
|  | S30 | gym* | [View Results](javascript:__doPostBack('ctl00$ctl00$MainContentArea$MainContentArea$historyControl$HistoryRepeater$ctl166$linkResults','')) (30,214) |
|  | S29 | fitness centre* | [View Results](javascript:__doPostBack('ctl00$ctl00$MainContentArea$MainContentArea$historyControl$HistoryRepeater$ctl167$linkResults','')) (469) |
|  | S28 | Fitness center* | [View Results](javascript:__doPostBack('ctl00$ctl00$MainContentArea$MainContentArea$historyControl$HistoryRepeater$ctl168$linkResults','')) (6,930) |
|  | S27 | cycling | [View Results](javascript:__doPostBack('ctl00$ctl00$MainContentArea$MainContentArea$historyControl$HistoryRepeater$ctl169$linkResults','')) (54,264) |
|  | S26 | squash | [View Results](javascript:__doPostBack('ctl00$ctl00$MainContentArea$MainContentArea$historyControl$HistoryRepeater$ctl170$linkResults','')) (2,345) |
|  | S25 | table tennis | [View Results](javascript:__doPostBack('ctl00$ctl00$MainContentArea$MainContentArea$historyControl$HistoryRepeater$ctl171$linkResults','')) (2,713) |
|  | S24 | lacrosse | [View Results](javascript:__doPostBack('ctl00$ctl00$MainContentArea$MainContentArea$historyControl$HistoryRepeater$ctl172$linkResults','')) (7,164) |
|  | S23 | shooting | [View Results](javascript:__doPostBack('ctl00$ctl00$MainContentArea$MainContentArea$historyControl$HistoryRepeater$ctl173$linkResults','')) (12,634) |
|  | S22 | equestrian | [View Results](javascript:__doPostBack('ctl00$ctl00$MainContentArea$MainContentArea$historyControl$HistoryRepeater$ctl174$linkResults','')) (5,463) |
|  | S21 | archery | [View Results](javascript:__doPostBack('ctl00$ctl00$MainContentArea$MainContentArea$historyControl$HistoryRepeater$ctl175$linkResults','')) (5,484) |
|  | S20 | (motorcycl* or motor cycl*) | [View Results](javascript:__doPostBack('ctl00$ctl00$MainContentArea$MainContentArea$historyControl$HistoryRepeater$ctl176$linkResults','')) (25,241) |
|  | S19 | ((motor or auto) n1 sport*) | [View Results](javascript:__doPostBack('ctl00$ctl00$MainContentArea$MainContentArea$historyControl$HistoryRepeater$ctl177$linkResults','')) (18,551) |
|  | S18 | ((horse or harness or dog or motor or auto or car) n1 rac*) | [View Results](javascript:__doPostBack('ctl00$ctl00$MainContentArea$MainContentArea$historyControl$HistoryRepeater$ctl178$linkResults','')) (16,143) |
|  | S17 | bowling | [View Results](javascript:__doPostBack('ctl00$ctl00$MainContentArea$MainContentArea$historyControl$HistoryRepeater$ctl179$linkResults','')) (11,621) |
|  | S16 | lawn bowls | [View Results](javascript:__doPostBack('ctl00$ctl00$MainContentArea$MainContentArea$historyControl$HistoryRepeater$ctl180$linkResults','')) (1,050) |
|  | S15 | sailing | [View Results](javascript:__doPostBack('ctl00$ctl00$MainContentArea$MainContentArea$historyControl$HistoryRepeater$ctl181$linkResults','')) (6,789) |
|  | S14 | boating | [View Results](javascript:__doPostBack('ctl00$ctl00$MainContentArea$MainContentArea$historyControl$HistoryRepeater$ctl182$linkResults','')) (7,889) |
|  | S13 | rowing | [View Results](javascript:__doPostBack('ctl00$ctl00$MainContentArea$MainContentArea$historyControl$HistoryRepeater$ctl183$linkResults','')) (8,976) |
|  | S12 | yacht* | [View Results](javascript:__doPostBack('ctl00$ctl00$MainContentArea$MainContentArea$historyControl$HistoryRepeater$ctl184$linkResults','')) (18,768) |
|  | S11 | handball* | [View Results](javascript:__doPostBack('ctl00$ctl00$MainContentArea$MainContentArea$historyControl$HistoryRepeater$ctl185$linkResults','')) (10,147) |
|  | S10 | australian rules football* | [View Results](javascript:__doPostBack('ctl00$ctl00$MainContentArea$MainContentArea$historyControl$HistoryRepeater$ctl186$linkResults','')) (496) |
|  | S9 | (waterski* or water ski*) | [View Results](javascript:__doPostBack('ctl00$ctl00$MainContentArea$MainContentArea$historyControl$HistoryRepeater$ctl187$linkResults','')) (2,471) |
|  | S8 | (waterpolo or water polo) | [View Results](javascript:__doPostBack('ctl00$ctl00$MainContentArea$MainContentArea$historyControl$HistoryRepeater$ctl188$linkResults','')) (2,482) |
|  | S7 | triathl* | [View Results](javascript:__doPostBack('ctl00$ctl00$MainContentArea$MainContentArea$historyControl$HistoryRepeater$ctl189$linkResults','')) (17,716) |
|  | S6 | softball* | [View Results](javascript:__doPostBack('ctl00$ctl00$MainContentArea$MainContentArea$historyControl$HistoryRepeater$ctl190$linkResults','')) (5,227) |
|  | S5 | canoe* | [View Results](javascript:__doPostBack('ctl00$ctl00$MainContentArea$MainContentArea$historyControl$HistoryRepeater$ctl191$linkResults','')) (8,096) |
|  | S4 | rugby | [View Results](javascript:__doPostBack('ctl00$ctl00$MainContentArea$MainContentArea$historyControl$HistoryRepeater$ctl192$linkResults','')) (52,067) |
|  | S3 | netball* | [View Results](javascript:__doPostBack('ctl00$ctl00$MainContentArea$MainContentArea$historyControl$HistoryRepeater$ctl193$linkResults','')) (1,287) |
|  | S2 | cricket | [View Results](javascript:__doPostBack('ctl00$ctl00$MainContentArea$MainContentArea$historyControl$HistoryRepeater$ctl194$linkResults','')) (9,178) |
|  | S1 | sport* | (845,911) |

**COCHRANE LIBRARY**

**Cochrane Reviews – 12 (new-9)**

**CENTRAL – 135 (new-19)**

**Not Related**

'(Sport* or cricket* or netball* or rugby* or canoe* or softball* or triathl* or waterpolo or "water polo" or waterski or "water ski*" or "Australian rules football*" or surfing or handball* or yacht* or rowing or boating or sailing or bowl* or "horse rac*" or "harness rac*" or "dog rac*" or "motor rac*" or "auto rac*" or "car rac*" or "motor sport*" or "auto sport*" or archery or equestrian or shooting or hunting or lacrosse or polo or "table tennis" or badminton or squash or cycling or "fitness center*" or "fitness centre*" or gym* or (sport* and (game* or event* or club* or arena* or field* or ground*)) or player* or athlet* or spectator* or fan* or (sport* and member*)) in Title, Abstract, Keywords and ("health promotion" or "public health" or "harm reduction" or "harm minimi*" or "health policy" or "public policy" or program or intervention* or "preventive medicine" or "health education" or "consumer health information" or "patient education" or environment* or (responsible and (alcohol* or beverage*) and servic*) or "server training" or "server intervention*" or enforcement or "community action*" or "community mobili*" or (alcohol* and control*) or strateg*) in Title, Abstract, Keywords and (Alcohol* or "drunk* driv*" or "drink* driv*") in Title, Abstract, Keywords and (Trial* or randomi* or "double blind" or "single blind" or experiment* or pretest or "pre test" or posttest or "post test" or "pre post" or prepost or "before after" or "quasi randomised" or "quasi randomized" or "quazi randomised" or "quazi randomized" or "stepped wedge" or "non randomized" or "non randomised" or nonrandomized or nonrandomised or "time series" or "multiple baseline" or "regression discontinuity" or "comprehensive cohort")

**DISSERTATIONS AND THESES – 119 (duplicates included)**

**3 new but unrelated**

**ERIC – 17 (but only 1 after April 2014- unrelated)**

(Sport* or cricket* or netball* or rugby* or canoe* or softball* or triathl* or waterpolo or “water polo” or waterski or “water ski*” or “Australian rules football*” or surfing or handball* or yacht* or rowing or boating or sailing or bowl* or “horse rac*” or “harness rac*” or “dog rac*” or “motor rac*” or “auto rac*” or “car rac*” or “motor sport*” or “auto sport*” or archery or equestrian or shooting or hunting or lacrosse or polo or “table tennis” or badminton or squash or cycling or “fitness center*” or “fitness centre*” or gym* or (sport* and (game* or event* or club* or arena* or field* or ground*)) or player* or athlet* or spectator* or fan* or (sport* and member*))

AND

(“health promotion” or “public health” or “harm reduction” or “harm minimi*” or “health policy” or “public policy” or program or intervention* or “preventive medicine” or “health education” or “consumer health information” or “patient education” or environment* or (responsible and (alcohol* or beverage*) and servic*) or “server training” or “server intervention*” or enforcement or “community action*” or “community mobili*” or (alcohol* and control*) or strateg*)

AND

(Alcohol* or “drunk* driv*” or “drink* driv*”)

AND

(Trial* or randomi* or “double blind” or “single blind” or experiment* or pretest or “pre test” or posttest or “post test” or “pre post” or prepost or “before after” or “quasi randomised” or “quasi randomized” or “quazi randomised” or “quazi randomized” or “stepped wedge” or “non randomized” or “non randomised” or nonrandomized or nonrandomised or “time series” or “multiple baseline” or “regression discontinuity” or “comprehensive cohort”)
