# Supplementary figures and images for: Interventions in sports settings to reduce risky alcohol consumption and alcohol-related harm: a systematic review
Source: Syst Rev. 2016 Jan 21;5:12. doi: 10.1186/s13643-016-0183-y (PMC4721008; doi:10.1186/s13643-016-0183-y)

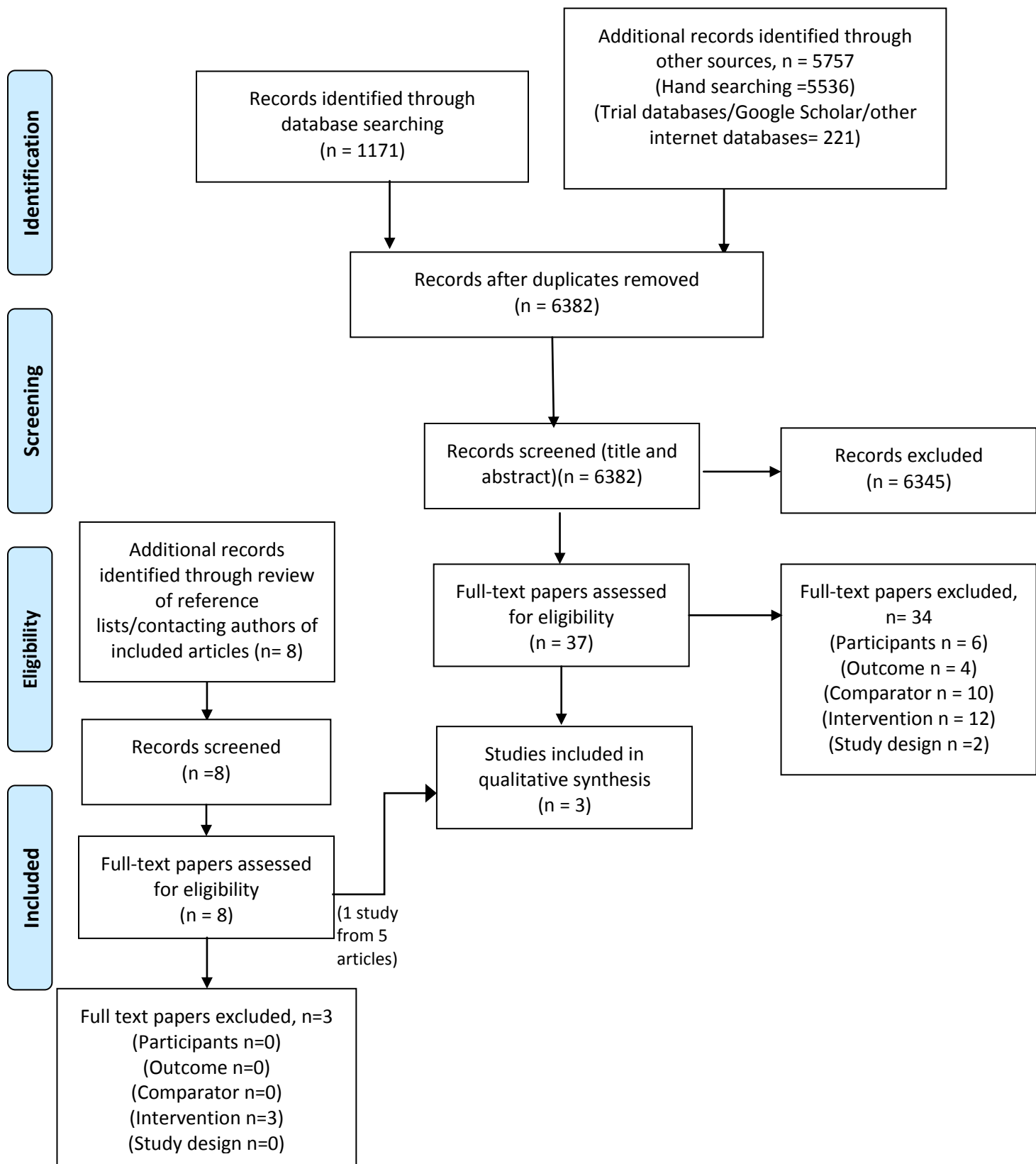

Supplement: Additional file 2: — PRISMA flowchart. This file contains the PRISMA flowchart for the review. The flowchart includes the number of studies screened, number of studies excluded and number of studies included in the review.(PDF 190 kb) [file 13643_2016_183_MOESM2_ESM.pdf]
