# Supplementary material for: Interventions in sports settings to reduce risky alcohol consumption and alcohol-related harm: a systematic review
Source: Syst Rev. 2016 Jan 21;5:12. doi: 10.1186/s13643-016-0183-y (PMC4721008; doi:10.1186/s13643-016-0183-y)
Supplement: Additional file 3: — Risk of bias assessment: detailed results. This file contains the detailed results of the risk of bias assessment of included studies. The reasons for each assessment rating are provided.(DOCX 21 kb) [file 13643_2016_183_MOESM3_ESM.docx]

| **Risk of bias** | **Description** | **Judgment** |
| --- | --- | --- |
| ***Carr 1992 (Overall risk of bias: HIGH)*** | | |
| Sequence generation (Selection bias) | The procedure for generating the randomisation sequence is not described. | Unclear |
| Allocation concealment (Selection bias) | No information is reported on whether there was potential for prior knowledge of upcoming allocation. | Unclear |
| Blinding of participants and personnel (Performance bias) | Not possible to blind participants or personnel due to nature of the intervention**.** Each team was contacted and athletes told of their condition assignment prior to pre-test. | High |
| Blinding of outcome assessor (Self-reported outcomes) | Self-reported outcomes collected via self-administered questionnaires and therefore not possible to blind outcome assessors. | High |
| Incomplete outcome data  (Self-reported outcomes) | 17 of 70 participants did not complete post-intervention or the follow-up assessments. They were not included in the analysis. | High |
| Selective reporting bias | While not all outcome data were fully reported (missing frequency counts), provision of the results of the outcome analysis suggests that there is unlikely to be bias due to selective reporting. | Low |
| Other sources of bias | None identified. | Low |
| ***Kingsland 2015 (Overall risk of bias: LOW)*** | | |
| Sequence generation (Selection bias) | Microsoft Excel random-number generator using simple randomisation 1:1 ratio. | Low |
| Allocation concealment (Selection bias) | Central, computerised allocation was undertaken for all participants at once. Allocation was carried out by an independent statistician. | Low |
| Blinding of participants and personnel (Performance bias) | Study personnel were not blind to allocation. Not possible to blind participating clubs or club members to allocation. | High |
| Blinding of outcome assessor (Self-reported outcomes) | Research personnel involved in post-intervention data collection (telephone survey) and analysis were blind to allocation. While club members who self-reported study outcomes were not intentionally blinded from knowing which group their club was allocated to, their knowledge of this is unknown. | Unclear |
| Incomplete outcome data  (Self-reported outcomes) | All data were accounted for in the analysis. Intention to treat analysis was undertaken. | Low |
| Selective reporting bias | All pre-specified study outcomes as outlined in the study protocol were reported and explanation was provided for an outcome (7-day diary) that was not reported (found to be unsuitable for study context due to members not consuming alcohol at their club on a weekly basis). | Low |
| Other sources of bias (participant selection in a cluster trial) | Participants for both groups were selected based on a quasi-random procedure based on birth date. | Low |
| ***O’Farrell 2010 (Overall risk of bias: HIGH)*** | | |
| Sequence generation (Selection bias) | No sequence generation - closest county was selected for intervention. | High |
| Allocation concealment (Selection bias) | Unconcealed procedure as selection of control and intervention sites was based on convenience. | High |
| Blinding of participants and personnel (Performance bias) | Study personnel were not blind to allocation. Not possible to blind participating clubs or players to allocation. | High |
| Blinding of outcome assessor (Self-reported outcomes) | Players who self-reported study outcomes (via self-administered questionnaires) were aware of their club’s allocation. | High |
| Incomplete outcome data  (Self-reported outcomes) | The study did not address this issue. | Unclear |
| Selective reporting bias | There is no indication that any relevant outcomes were not reported. | Low |
| Other sources of bias (participant selection in a cluster trial) | For both groups, all eligible club members were invited to participate. There was no selection process. | Low |
